# Lewis Acid‐Activated Charge Trapping in Dielectric Polymers for Superior High‐Temperature Electrostatic Energy Storage

**DOI:** 10.1002/advs.202517934

**Published:** 2025-11-19

**Authors:** Lu Fan, Zongliang Xie, Xi Chen, Qingsong Zhang, Yalin Wang, He Li, Xi Pang, Tiffany Chen, Shiqi Lai, Zhiyuan Huang, Ashlin M. Deatherage, Hanjiang Gu, Meng Chen, Tao Han, Liana M. Klivansky, Steve W. Shelton, Peng Liu, Zongren Peng, Ting Xu, Jian Zhang, Yi Yin, Yi Liu

**Affiliations:** ^1^ The Molecular Foundry Lawrence Berkeley National Laboratory Berkeley CA 94720 USA; ^2^ School of Electrical Engineering Shanghai Jiao Tong University Shanghai 200240 China; ^3^ Materials Sciences Division Lawrence Berkeley National Laboratory Berkeley California 94720 USA; ^4^ School of Materials Science and Engineering Nanyang Technological University Singapore 639798 Singapore; ^5^ State Key Laboratory of Electrical Insulation and Power Equipment Xi'an Jiaotong University Xi'an Shaanxi 710049 China; ^6^ Department of Chemistry University of California Berkeley California 94720 USA; ^7^ School of Automation and Intelligent Sensing Shanghai Jiao Tong University Shanghai 200240 China; ^8^ Department of Materials Science and Engineering University of California Berkeley CA 94720 USA

**Keywords:** charge dynamics, energy storage, lewis acid, polymer dielectrics

## Abstract

Dielectric polymer capacitors are essential for electrostatic energy storage but suffer from charge transport‐induced energy losses, particularly at elevated temperatures where thermally activated charge carriers exacerbate conduction. Conventional mitigation strategies rely on introducing heterogeneous interfaces to create charge traps, complicating scalable film fabrication. A homogeneous molecular trapping mechanism would circumvent these complexities, yet remains underexplored. Herein, a charge trapping strategy is devised by modifying the lowest occupied molecular orbitals of dielectric polymers through Lewis acid‐base adduct formation. The use of tris(pentafluorophenyl)boron (BCF) as a Lewis acidic molecular additive introduces deeper charge traps in commercial polyetherimide (PEI) while retaining homogeneity. With only 0.5 wt.% loading, the PEI‐BCF film exhibits greatly improved breakdown strength, achieving an ultrahigh discharged energy density of 7.3 J cm^−3^ with excellent cycle stability at 200 °C. This work establishes a facile molecular approach to decoupling charge trapping from heterogeneous interfaces, enabling high‐energy‐density polymer capacitors operable under extreme thermal conditions.

## Graphical abstract

Lewis acid–base molecular engineering tailors the lowest unoccupied molecular orbital of polymers to form deep, homogeneous charge traps in commercial polyetherimide (PEI). These deep traps effectively inhibit the transport of injected charge carriers from electrodes, ultimately enabling ultrahigh energy density and thermal stability in dielectric polymer capacitors.

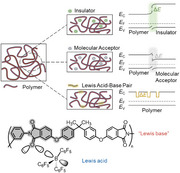

## 1 Introduction

Dielectric polymers are indispensable electrostatic energy storage materials extensively utilized in modern film capacitors, owing to their high breakdown strength, light weight, excellent insulating properties, and great mechanical flexibility.^[^ ^1^, ^2^ ^]^ However, their performance degrades drastically at operational temperatures exceeding 150–200 °C due to thermally accelerated charge transport, which elevates leakage current (*J*) and reduces electric field tolerance (i.e., breakdown strength, *E*_b_). Concurrently, critical energy storage metrics, such as discharged energy density (*U*_d_) and charge–discharge efficiency (*η*), are significantly compromised.^[^ ^3^, ^4^, ^5^, ^6^ ^]^ This thermal intolerance severely limits their use in high‐temperature applications essential to electrified transportation, aerospace systems, renewable energy platforms, and underground exploration.^[^ ^2^, ^7^, ^8^, ^9^, ^10^ ^]^ One feasible approach to addressing this challenge involves developing innovative strategies to enhance charge carrier trapping, thereby improving energy storage performance while retaining the intrinsic advantages of dielectric polymers.^[^ ^11^, ^12^, ^13^, ^14^ ^]^

**Figure 1:**
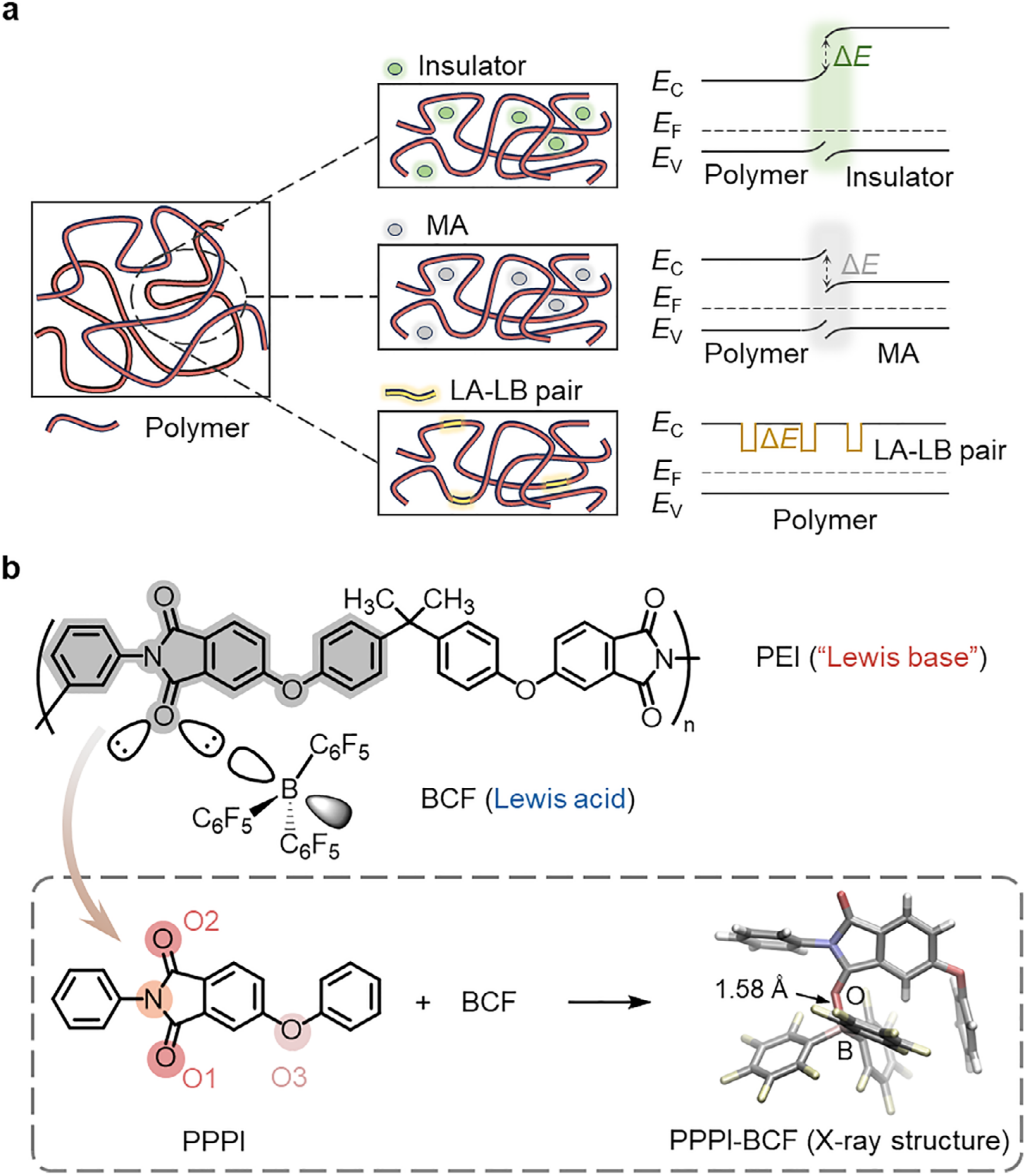
a) Illustration of different charge‐trapping mechanisms in dielectric polymer‐based composites. Green shadow represents the interface between polymer and insulator, gray shadow represents the interface between polymer and molecular acceptor (MA), and yellow wells represent mid‐gap traps formed via Lewis acid‐Lewis base (LA‐LB) pair. b) Formation of Lewis acid‐base pair between polyetherimide (PEI) and tris(pentafluorophenyl)borane (BCF). PEI is not known as a Lewis base but acts as one when pairing with BCF, thus the quotation marks. Single‐crystal XRD characterization of the complex formed between BCF and the molecular analogy, PPPI, with BCF binding favorably to the imido O1 atom. Red‐colored circles represent imido Oxygen (O1 and O2), pink‐colored circle represents ether Oxygen (O3), and orange‐colored circle represents imide Nitrogen. In X‐ray structure, white color represents Hydrogen atoms, pink color represents Boron atoms, gray color represents Carbon atoms, red color represents Oxygen atoms, purple color represents Nitrogen atoms, and yellow color represents Fluorine atoms.

Various additives have been explored extensively to trap injected and thermally excited charge carriers.^[^ ^10^, ^15^, ^16^, ^17^, ^18^ ^]^ Conventional inorganic and hybrid nanofillers, such as boron nitride nanosheets,^[^ ^19^ ^]^ self‐assembled supramolecular nanosheets,^[^ ^20^, ^21^ ^]^ polymer‐grafted nanoparticles,^[^ ^22^ ^]^ oxides,^[^ ^23^ ^]^ 2D subnanosheets,^[^ ^24^ ^]^ multilayered nanolaminates,^[^ ^25^ ^]^ and metal–organic frameworks,^[^ ^26^ ^]^ typically rely on charge immobilization at polymer‐filler interfaces (**Figure** **1**a). Despite their potential, these heterogeneous interfaces often incur complications such as poor solubility, nonuniform dispersion, and agglomeration, significantly undermining the utility of inorganic fillers in scalable fabrication of composite films.^[^ ^10^, ^27^, ^28^ ^]^ An alternative strategy employs molecular additives to introduce electron traps at polymer‐molecule interfaces, where electron‐deficient organic semiconductors serve as electron traps owing to their intrinsic electron affinity or through charge‐transfer interactions with the polymer matrix (Figure 1a).^[^ ^29^, ^30^ ^]^ Nevertheless, the effectiveness of this method is constrained by the electron affinity of available electron acceptors and their tendency to increase conductivity due to their high carrier mobilities. A molecular trapping strategy independent of interfaces and heterogeneity, thus, remains highly desirable.

In this work, we introduce a fundamentally distinct molecular trapping strategy centered on inducing mid‐gap electron traps within dielectric polymers through the formation of a Lewis acid‐Lewis base adduct (Figure 1a). As demonstrated in the system involving polyetherimide (PEI) as the Lewis base and tris(pentafluorophenyl)borane (BCF) as the Lewis acid, B•••O interactions between the imido oxygen atoms of PEI and the boron centers of BCF substantially alter PEI's electronic structures. Unlike conventional π‐acidic electron acceptor‐based molecular traps,^[^ ^29^, ^30^ ^]^ the Lewis acid method directly lowers the polymer's lowest unoccupied molecular orbital (LUMO) energy level, resulting in increased electron affinity and deep trap states that efficiently immobilize charge carriers. Films based on the PEI‐BCF complex exhibit remarkably suppressed conduction losses and exceptional thermal resilience, achieving an ultrahigh discharged energy density of 7.3 J cm^−3^ and charge–discharge cycle stability over 5 × 10^5^ cycles at 200 °C. This work establishes a conceptual platform for efficient charge trapping while bypassing interfacial heterogeneity, advancing the design of high‐temperature dielectric polymers for next‐generation energy storage systems.

## 2 Results and Discussion

### 2.1 Establishing BCF‐PEI Interactions

BCF is a versatile Lewis acid with excellent solubility and thermal stability,^[^ ^31^, ^32^ ^]^ widely used in organic synthesis^[^ ^31^, ^33^ ^]^ and doping organic semiconductors.^[^ ^34^, ^35^, ^36^, ^37^ ^]^ Its empty boron‐centered π‐orbital, together with strongly electron‐withdrawing perfluorinated phenyl groups, confers high affinity toward Lewis‐basic atoms such as oxygen and nitrogen.^[^ ^36^, ^38^, ^39^, ^40^, ^41^ ^]^ The repeat unit of PEI offers multiple potential Lewis basic coordination sites, including etheric and imido oxygens and nitrogen atom in the imide group (Figure 1b). We envision that BCF interacts with imido groups through dative B•••O bond formation, serving as a foundational mechanism to modulate the electronic properties of PEI for charge trapping. Such interactions were evidenced by single‐crystal X‐ray diffraction (SCXRD) studies (see Experimental Section) of the adduct formed between BCF and 4‐phenoxy‐*N*‐phenyl‐phthalimide (PPPI) (Note S1 and Figures S1 and S2, Supporting Information), a molecular analog that resembles the Lewis basic segment of PEI (Figure 1b). The X‐ray structure of the obtained single crystals revealed 1:1 complexation between BCF and PPPI, with boron preferentially coordinating with O1 of the imide moiety, exhibiting a B•••O distance of 1.58 Å (Figure S3 and Table S1, Supporting Information). Density functional theory (DFT) calculations based on the single‐crystal structure revealed that this B•••O coordination significantly lowered PPPI's LUMO energy from −2.48 to −3.80 eV (Figures S4 and S5, Supporting Information). A yellow color was developed upon mixing the colorless solutions of BCF and PPPI (Figure S6, Supporting Information), in accordance with the coordination‐modulated reduction of the optical gap. Similar color change and 1:1 adduct formation were also observed between BCF and the phenoxy‐free phthalimide molecular analog, *N*‐phenylphthalimide (PID), where a comparable B•••O distance (1.58 Å) was observed in its crystal structure (Figures S6 and S7 and Table S2, Supporting Information). DFT calculations of the PEI‐BCF complex indicated preferential BCF coordination with imido oxygen atoms, characterized by a short B•••O distance of 1.60 Å (Figure S8, Supporting Information), in agreement with the B•••O distance observed in the single crystal structure of PPPI‐BCF (Figure 1b; Figure S3, Supporting Information). This B•••O coordination localized the LUMO orbital near the coordination sites and similarly lowered PEI's LUMO energy from −2.64 to −3.64 eV, effectively engendering higher electron demand while showing minimal effect on its HOMO orbital (Figures S4,S9, and S10, Supporting Information). Both the crystal structure of the PPPI‐BCF adduct and the calculation results provided compelling evidence for the preferential coordination of BCF with the imide oxygen atoms in PEI via Lewis acid‐base interactions. Moreover, the calculated binding energies of DFT‐optimized PPPI‐BCF and PEI‐BCF structures are −48.23 and −34.06 kcal•mol^−1^, respectively, providing further evidence of the strong interaction between B and O. Atomic dipole corrected Hirshfeld (ADCH) calculations (see Experimental Section) revealed approximately 0.4 electron transfer from imido oxygen to boron upon coordination (Figure S11, Supporting Information), with diminishing degree of electron transfer further along the polymer backbone.

**Figure 2:**
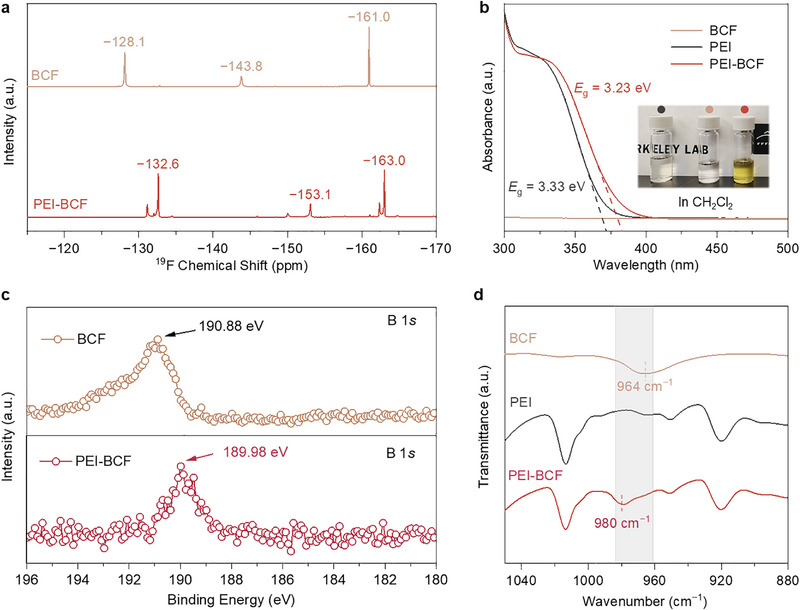
a) ^19^F NMR spectroscopy of BCF and PEI‐BCF complex in solution (470 MHz, CD_2_Cl_2_, 298 K). b) UV–vis spectra of PEI, BCF, and PEI‐BCF complex (in CH_2_Cl_2_); the inset shows the visual color of the solutions. c) XPS spectra of B1s peaks of BCF and PEI‐BCF complex. d) FTIR spectroscopy of PEI, BCF, and PEI‐BCF complex.

NMR (^19^F and ^13^C) spectroscopic results further confirmed Lewis acid‐base interactions in CD_2_Cl_2_ solution (**Figure** **2**a; Figure S12, Supporting Information). Upfield shifts of fluorine resonances in BCF and downfield shifts of carbonyl carbon resonances in PEI indicated electron transfer to BCF upon B•••O coordination, aligning with simulation results (Figure S11, Supporting Information). Similar to the cases of BCF‐PPPI and BCF‐PID adducts, a yellow color was developed upon mixing the colorless solutions of BCF and PEI, confirming BCF‐PEI interactions that reduce PEI's optical gap (Figure 2b; Figure S4, Supporting Information). Such interactions persisted in thin films, as verified by various spectroscopic studies. X‐ray photoelectron spectroscopy (XPS) of PEI‐BCF films revealed shifts in binding energies for boron atoms (190.88 to 189.98 eV) and carbonyl oxygen atoms (531.23 to 531.45 eV), consistent with B•••O binding (Figure 2c; Figure S13, Supporting Information). Fourier‐transfer infrared (FTIR) spectra revealed a characteristic B‐C stretching vibration peak at 964 cm^−1^ for the PEI‐BCF film (at 5 wt.% BCF loading) compared to 980 cm^−1^ for pure BCF (Figure 2d). Those results confirmed the persistence of B•••O interactions throughout the high‐temperature film formation process. Additional studies on morphology, glass transition temperature, and mechanical properties revealed negligible variations between pure PEI and PEI‐BCF films, indicating that the additive has a negligible impact on film formation and fundamental physical properties (Figures S14–S16, Supporting Information).

### 2.2 Dielectric Properties and Charge Transport Behavior

**Figure 3:**
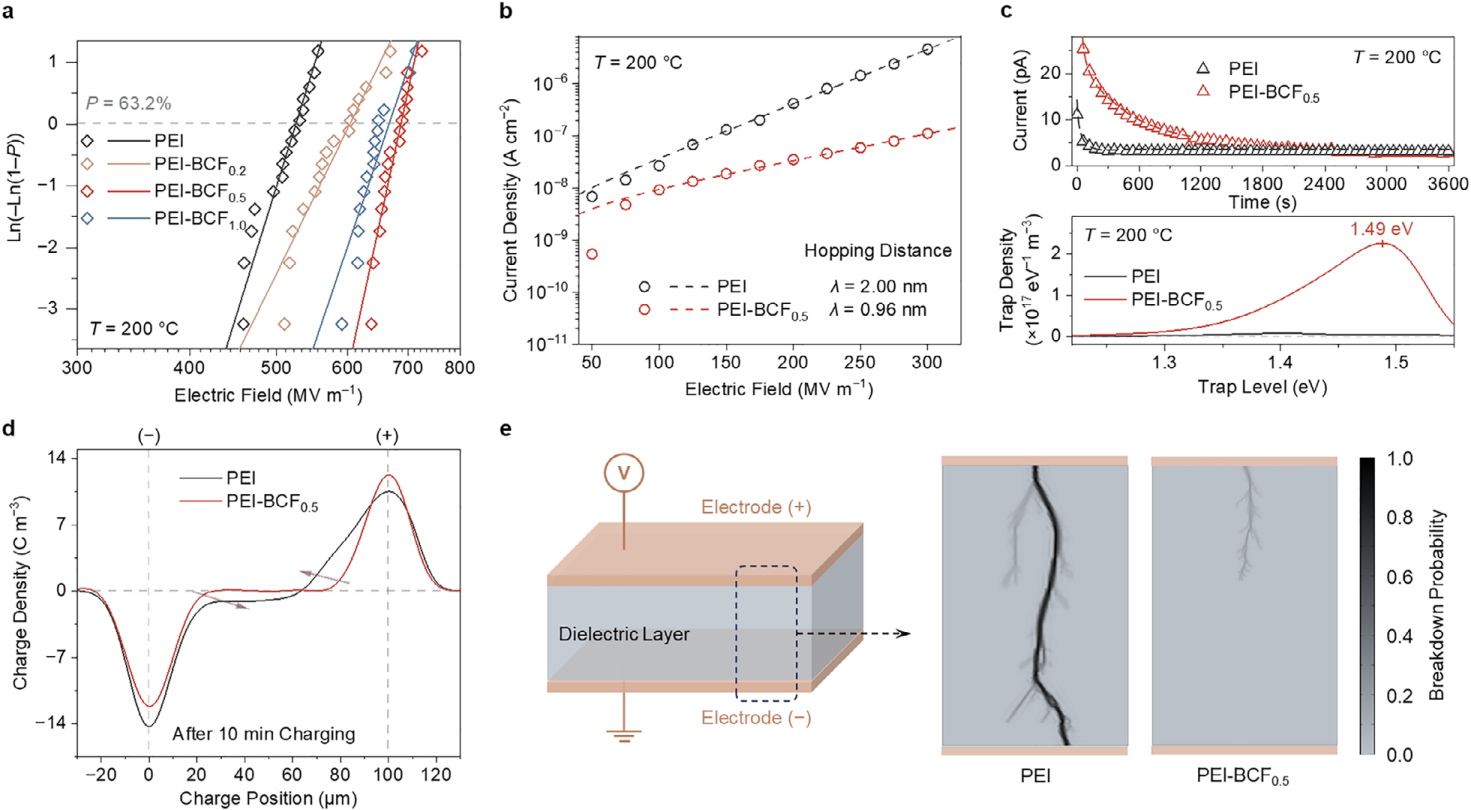
a) Weibull breakdown plots of pure PEI and PEI‐BCF_n_ at 200 °C, with a sampling size of 16. b) Hopping conduction fittings of leakage current density versus electric field of pure PEI and PEI‐BCF_0.5_, measured at 200 °C. c) Isothermal relaxation current of PEI and PEI‐BCF_0.5_ measured after polarization under 100 MV m^−1^ for 10 min at 200 °C (top), along with trap density and energy level calculated from IRC results (bottom). d) Space charge density distribution of PEI and PEI‐BCF_0.5_, see Experimental Section. e) Simulated electrical tree propagation of PEI and PEI‐BCF_0.5_.

The dielectric strength of PEI‐BCF films was derived from measurement of dielectric breakdown strength (*E*_b_) and leakage current density (*J*). Films containing different BCF loadings were denoted as PEI‐BCF_n_ (where n = 0.2, 0.5, and 1 indicates BCF weight percentage, wt.%). The *E*_b_s of PEI‐BCF_n_ films at various temperatures were statistically analyzed using a two‐parameter Weibull distribution (see Experimental Section). All PEI‐BCF_n_ films showed higher Weibull *E*_b_s than pure PEI, along with high *β* values at 150 and 200 °C (**Figure** **3**a; Figure S17 and Table S3, Supporting Information), among which PEI‐BCF_0.5_ exhibited an optimal breakdown strength of 678 MV m^−1^ and *β* of 35 at 200 °C. Accordingly, PEI‐BCF_0.5_ displayed the smallest current density *J*, which was an order of magnitude lower than that of pure PEI (3.3 × 10^−8^ A cm^−2^ *vs* 4.2 × 10^−7^ A cm^−2^ at 200 °C and 200 MV m^−1^, see Figure 3b; Figure S18, Supporting Information). Given that current leakage is a key pathway of energy loss in dielectric materials, the smallest *J* observed in PEI‐BCF_0.5_ at elevated temperatures and high electric fields alluded to its superior energy density and efficiency. Analysis of the current density‐electric field (*J*‐*E*) relationship was conducted by fitting the data to the hopping conduction equation (see Experimental Section). The hopping distance in PEI was higher compared with that of the PEI‐BCF_n_ composites. Among these, PEI‐BCF_0.5_ displayed a hopping distance of 0.96 nm, in contrast to 2 nm for pure PEI. As the hopping distance indicates the average spacing between trap sites, the smaller value observed in PEI‐BCF_0.5_ reflects a larger density of trap sites, supporting its better charge capture capabilities. In addition to enhancing the electrical strength, the introduction of BCF exhibited minimal influence on dielectric constant and dielectric loss of PEI, as shown by the frequency‐ and temperature‐dependent dielectric spectroscopy of PEI‐BCF_n_ films (Figure S19, Supporting Information).

The charge carriers injected from the electrodes are the primary source of conductivity in insulating polymers under high temperature and high electric field.^[^ ^30^, ^44^, ^45^ ^]^ Deep traps introduced within the PEI chains through Lewis acid‐base coordination between BCF and PEI serve as a key factor in inhibiting the transport of injected charge carriers. We evaluated the charge trapping behavior through isothermal relaxation current (IRC, see Experimental Section^[^ ^42^ ^]^). IRC curve of PEI‐BCF_0.5_ film exhibited a slower decay rate compared to that of PEI film, indicating that trapped charges in PEI‐BCF_0.5_ required a longer time to be released (Figure 3c). Analysis based on the trap density extraction method^[^ ^42^ ^]^ revealed that the PEI‐BCF_0.5_ film possessed deeper trap depth (1.49 eV) and trap density (2.25 × 10^17^ cm^−3^) compared to pure PEI. Similar conclusions were supported by thermally stimulated depolarization current (TSDC) analyses, where PEI‐BCF_0.5_ exhibited a larger depolarization current peak and a new shoulder peak at a higher temperature (Figure S20, Supporting Information).

In line with the presence of deep traps in PEI‐BCF_0.5_, injected homo charges can be effectively captured near the electrode‐film interfaces, resulting in a built‐in reverse field that further inhibits subsequent charge injection from the electrodes into the internal regions of the film. This phenomenon was verified by pulsed electro‐acoustic (PEA) measurements, which revealed negligible accumulation of space charge within PEI‐BCF_0.5_. In contrast, significant charge accumulation was observed in pure PEI (Figure 3d, see Experimental Section), resulting in a large electric field distortion with the maximum internal electric field exceeding the applied field by 35% (Figure S21, Supporting Information), which adversely affected the dielectric breakdown strength. Based on the trap properties obtained from IRC results, numerical simulation using a modified 2D bipolar charge transport‐dielectric breakdown (2D BCT‐DB) model (see Experimental Section, Note S2 and Tables S4 and S5, Supporting Information) indicated that, in contrast to PEI where electrical treeing propagated to complete breakdown, deep traps in PEI‐BCF_0.5_ effectively suppressed charge accumulation and migration (Figure S22, Supporting Information), which significantly reduced electric field distortion and retarded treeing growth, enabling the retention of insulation strength under the same electric field (Figure 3e).

### 2.3 Energy Storage and Reliability Performance

**Figure 4:**
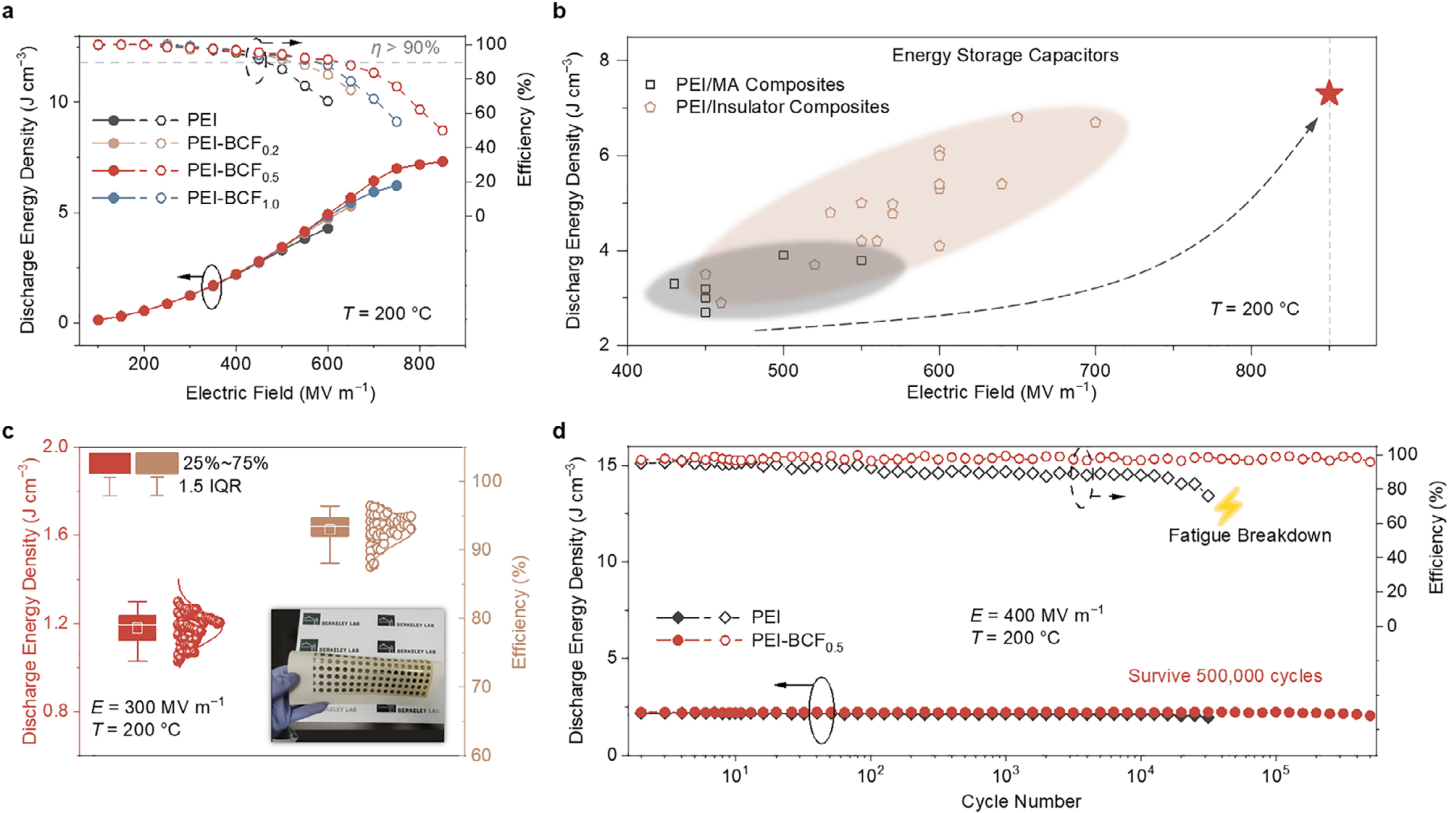
a) Discharged energy density and charge–discharge efficiency of pure PEI and PEI‐BCF_n_ at 200 °C. b) Comparison of the maximum discharged energy density of the PEI‐BCF_0.5_ composite against high‐temperature PEI‐based composites at 200 °C. c) IQR analysis of energy storage characteristics of an array of 60 devices fabricated on a large‐area PEI‐BCF_0.5_ film, measured at 200 °C and 300 MV m^−1^. d) Cycling stability of devices based on PEI‐BCF_0.5_ and PEI, measured at 200 °C and 400 MV m^−1^.

Breakdown strength and electrical conduction characteristics are key factors in determining energy storage performance. The breakdown strength determines the maximum discharge energy density according to the theoretical equation $U_{d}=\int_{D_{\max}}^{D_{\mathrm{rem}}}EdD\;$, wherein the maximum applied electric field *E* is determined by *E*_b_, the electric displacement *D* is positively correlated with both *E* and dielectric constant *κ*. In parallel, suppressing electrical conduction is vital for improving charge–discharge efficiency (*η*). The discharged energy density *U*_d_ and charge–discharge efficiency *η* of pure PEI and PEI‐BCF film capacitors were evaluated using unipolar electric displacement‐electric field (*D*‐*E*) loops. Consistent with suppressed leakage currents and reduced accumulation of space charges,^[^ ^43^, ^44^, ^45^ ^]^ energy loss was significantly reduced in PEI‐BCF_n_ films compared to pure PEI, as indicated by slimmer *D*‐*E* loops measured at 150 and 200 °C (Figure S23, Supporting Information). Among these films, PEI‐BCF_0.5_ exhibited the narrowest charging‐discharging loops. Under an extremely high field strength of 600 MV m^−1^, the charge–discharge efficiency *η* of pristine PEI decreased drastically to 67%, whereas PEI‐BCF_0.5_ maintained a notably high efficiency of 91.4% (**Figure** **4**a). Remarkably, PEI‐BCF_0.5_ sustained an extremely high electric field of 850 MV m^−1^ at 200 °C. Due to the enhanced *E*_b_ and significantly reduced energy loss (i.e., improved *η*), PEI‐BCF_n_ exhibited higher *U*_d_ compared to pristine PEI at both 150 and 200 °C, particularly at the optimal BCF loading of 0.5 wt.% (Figure 4a; Figure S24, Supporting Information). PEI‐BCF_0.5_ reached an outstanding maximum *U*_d_ of 7.3 J cm^−3^ at 200 °C and 8.9 J cm^−3^ at 150 °C, which was among the highest for PEI‐based composites (Figure 4b, data summarized in Table S6, Supporting Information). At efficiencies greater than 90%, PEI‐BCF_0.5_ achieved a maximum *U*_d_ of 5.3 J cm^−3^ at 200 °C and 6.8 J cm^−3^ at 150 °C, surpassing most known dielectric films (Table S6, Supporting Information). Furthermore, we investigated the performance of the PEI‐BCF_0.5_ film with a larger electrode area of 9 mm^2^. Notably, the film maintains an exceptionally high field strength of 600 MV m^−1^ with a charge–discharge efficiency of 90%, and the critical breakdown field strength remains at 800 MV m^−1^ (Figure S25, Supporting Information).

The high *η* ensures efficient energy utilization, essential for operational reliability and the longer lifespan of capacitor devices. To demonstrate the suitability for scaling up, PEI‐BCF_0.5_ films with a large area of 15 cm × 8 cm were fabricated using the same drop‐casting process. Reliability tests conducted at 300 MV m^−1^ and 200 °C on an array of 60 evenly distributed devices, at a larger electrode diameter of 6 mm, revealed consistently high efficiencies (medium *η* > 90%) through interquartile range (IQR) analysis (Figure 4c, see Experimental Section), attesting to the film's high uniformity and stability. During prolonged operation, the accumulated energy loss will cause gradual degradation of the polymer's insulation. The cyclic stability test reflects the film's fatigue properties over extended periods of operation. PEI‐BCF_0.5_ film maintained long‐term operational stability during further fatigue test at high temperature (Figure S26, Supporting Information). Owing to its low energy loss and high dielectric breakdown strength, both *U*_d_ and *η* of the PEI‐BCF_0.5_ film remained essentially unchanged after 5 00 000 cycles at 400 MV m^−1^ and 200 °C (Figure 4d). In contrast, PEI failed before 30 000 cycles under identical conditions, due to the accumulation of energy losses resulting from its low *η*.

## 3 Conclusion

BCF is known for its ability to boost the conductivity of semiconducting conjugated polymers by inducing the formation of charge carriers in the form of (di)polaron radicals, a process commonly referred to as doping.^[^ ^34^, ^35^, ^36^, ^37^ ^]^ Contradictory to the doping concept, we demonstrate here that by a different underlying principle, Lewis acidic molecular additives can effectively suppress charge transport in wide‐bandgap insulating polymers, rather than promote charge transport, as seen in medium‐to‐low bandgap semiconducting polymers. In polymers devoid of π‐basic conjugated ring systems, BCF's strong Lewis acidity specifically targets Lewis basic carbonyl oxygen atoms along the polymer backbone, resulting in effective charge trapping via the modification of its electronic structures. Such a mechanism is also supported by the correlation between Lewis acidity of different additives and *E*_b_ and *U*_d_ of PEI‐Lewis acid adduct films. For instance, employing a weaker Lewis acid such as triphenylboron (TPB, acceptor number 65.2 compared to 78.2 for BCF)^[^ ^46^, ^47^, ^48^ ^]^ yielded less pronounced enhancements in both *E*_b_ (624 MV m^−1^ *vs* 678 MV m^−1^) and overall energy storage performance (5.8 J cm^−3^ *vs* 7.3 J cm^−3^) at 200 °C (Table S7 and Figures S27 and S28, Supporting Information). Furthermore, incorporating just 0.5 wt.% BCF into other commercial carbonyl‐containing polymers (e.g., FPI, sPI, and FPE) utilized in high‐temperature capacitors led to enhanced energy storage performance at 200 °C compared to pristine polymers (Figures S29 and S30, Supporting Information), demonstrating the broad applicability of this approach.

As a wide bandgap molecular additive, a Lewis acid such as BCF does not rely on HOMO‐LUMO offsets as is the case for electron‐acceptor‐based additives, nor does it risk increasing conductivity like the latter. Additionally, its use as a nonintrusive, low‐percentage additive retains excellent film homogeneity of the pristine polymer via a solution process. These features collectively ensure a scalable process to obtain high‐quality dielectric films with exceptional thermal resilience, energy storage capacity, and long‐term cyclic reliability.

## 4 Experimental Section

### Single Crystal Preparation and Structural Characterization

All single‐crystal preparation procedures were carried out inside a glovebox. A mixture of PPPI or PID and BCF with a molar ratio of 1:1 was dissolved in CH_2_Cl_2_ at a concentration of 1 mmol mL^−1^. The resulting solution was transferred into a culture tube, after which *n*‐hexane was slowly added along the sidewall of the culture tube in a volume ratio of 1:4 (CH_2_Cl_2_:*n*‐hexane). After one week of standing, yellow crystals were obtained. Single crystal X‐ray diffraction data for PPPI‐BCF and PID‐BCF were collected at Beamline 12.2.1 of Advance Light Source, Lawrence Berkeley National Lab, Berkeley, CA. Indexing was performed using APEX5 (Difference Vectors method). Data integration and reduction were performed using SaintPlus 6.0. Absorption correction was performed by the multi‐scan method implemented in SADABS. Space groups were determined using XPREP implemented in APEX5. The structures were solved using SHELXS‐97 (direct methods) and refined using SHELXL‐97 within Olex 2 (full‐matrix least‐squares on F^2^). C, O, N, B, F atoms were refined with anisotropic displacement parameters, and H atoms were placed in geometrically calculated positions and included in the refinement process using a riding model with isotropic thermal parameters: *U*_iso_(H) = 1.2*U*_eq_(‐CH). Crystal data and refinement details are shown in Tables S1 and S2 (Supporting Information), and this data can be obtained free of charge from the Cambridge Crystallographic Data Centre (CCDC) via www.ccdc.cam.ac.uk/data_request/cif.

### Quantum Chemistry Calculation

All quantum chemical calculations were performed using the ORCA program.^[^ ^49^ ^]^ The molecular geometries were optimized at the B3LYP/def2‐SVP level of theory, followed by single‐point energy calculations employing the larger def2‐TZVP basis set. Dispersion corrections were accounted for using the DFT‐D3 method with Becke‐Johnson damping.^[^ ^50^ ^]^ Based on the calculated electronic structures, the atomic dipole moment corrected Hirshfield (ADCH) charge^[^ ^51^ ^]^ was calculated using the Multiwfn program^[^ ^52^ ^]^ to analyze the charge‐transfer between two adjacent molecules. Molecular structures and orbital visualizations were generated using the VMD program.^[^ ^53^ ^]^

### Electrical Characterization

Gold electrodes were sputtered on both sides of the polymeric films with a diameter of 6 mm for reliability and dielectric spectra testing. Frequency‐dependent and temperature‐dependent dielectric spectra over the frequency range between 100 Hz and 1 MHz and the temperature range from 20 to 200 °C of the samples were measured using an Agilent LCR meter (4294A), and a Delta Design 9023 oven was used to control the temperature. For large‐area film testing, devices located near the peripheral edges were excluded due to insufficient insulation spacing. Instead, 60 devices with an electrode diameter of 6mm in the central region, arranged in a 15 × 4 array, were selected for testing. Gold electrodes were sputtered on both sides of the polymeric films with a size of 1 mm × 1 mm and 3 mm × 3 mm for the DE loop and dielectric breakdown measurements. *D*‐*E* loops were collected using a modified Sawyer‐Tower circuit, where the samples were immersed in high‐temperature insulating fluid and subjected to a triangular unipolar wave with a frequency of 100 Hz. Dielectric breakdown strengths were measured with a DC ramp voltage of 500 V s^−1^ using a Trek 610E instrument as the voltage source. Gold electrodes were sputtered on both sides of the polymeric films with a size of 3 mm × 3 mm for the following measurements. Leakage current densities were acquired in a temperature chamber (EC1A, Sun Electronic Systems) using a Keithley 6514 electrometer coupled with an external Trek 610D amplifier as the voltage source. According to the hopping conduction equation, leakage current density (*J*_hopping_) is given as:

(1) $$J\left(E,T\right)=2ne\lambda v\times \exp\left(-\frac{W_{a}}{K_{B}T}\right)\times \sin h\left(\frac{\lambda eE}{2K_{B}T}\right)$$

where *E* is the applied electric field during current density measurement, *n* is the carrier concentration, *λ* is the hopping distance, *ν* is the attempt‐to‐escape frequency, *W*_a_ is the activation energy, *T* is the temperature, *e* is the charge of the carriers, *K*_B_ is the Boltzmann constant. Equation (1) can be simplified as:

(2) $$J\left(E\right)=A\times \sin h\left(B\times E\right)$$

where *A* and *B* are two lumped parameters. Both isothermal relaxation current (IRC) measurements and thermally stimulated depolarization current (TSDC) measurements were carried out in a Delta Design 9023 oven, and the current was measured using a Hewlett‐Packard 4140B pA meter. For IRC measurements, the samples were first polarized under an electric field of 100 MV m^−1^ at 200 °C for 10 min. After polarization, the samples were short‐circuited at 200 °C during which the depolarization current was recorded for 60 min. The trap depth (*E_t_*) was determined from measuring the time *t* using Equation (3):^[^ ^42^ ^]^

(3) $$E_{T}\left(t\right)=kT\ln\left(vt\right)$$

where *k* is the Boltzmann constant (8.617 × 10^−5^ eV K^−1^), *T* is the temperature (413.75 K), and *v* is the attempt‐to‐escape frequency, set as *kT*/*h* = 9.85 × 10^12^ s^−1^, where *h* is the Planck constant (4.1356 × 10^−15^ eV). The trap density was calculated from the relaxation current *I*(*t*) multiplied by time *t* using Equation (4):^[^ ^42^ ^]^

(4) $$N\left(E_{T}\right)=\frac{2I\left(t\right)t}{q\delta kTf_{0}\left(E\right)}$$

where *q* is the elementary charge, *δ* is the dielectric thickness, and *f*_0_(*E*) is the initial occupancy of the traps’ level, set as 0.5. For TSDC measurements, the samples were first polarized under an electric field of 100 MV m^−1^ at 220 °C for 10 min and then rapidly cooled down to 0 °C. The applied polarizing field was retained during the cooling process. The samples were kept at 0 °C for another 10 min with the polarizing field applied. After polarization, the samples were short‐circuited and heated to 260 °C at a rate of 3 °C min^−1^ during which the depolarization current was recorded. The space charge behavior of films was analyzed through the pulse electro‐acoustic (PEA) method with a nanosecond pulse generator.^[^ ^54^ ^]^ The pulsed electric field induced the vibration of space charges within the material, generating acoustic waves that propagate to a piezoelectric sensor—specifically, a PVDF‐TrFE (80/20) film with a thickness of 4.8 µm—enabling the reconstruction of the space charge distribution. The PEA testing film with a thickness of ≈100 µm was polarized under an electric field of 20 MV m^−1^ at 100 °C for 10 min. The internal electric field distribution of the film was subsequently calculated based on the space charge distribution using Poisson's equation.

### Finite Element Simulation

A bipolar charge transport dielectric breakdown (BCT‐DB) model was used to simulate time‐dependent charge behavior with the trap properties obtained from IRC results, electric field distributions and dielectric breakdown propagation paths, by solving partial differential equations (current continuity equation, the Poisson equation and the transport equation) using COMSOL Multiphysics version 6.0 with MUMPS and conjugate solver, detailed information see Note S2 (Supporting Information).

### Statistical Analysis

The breakdown strength was evaluated by performing a two‐parameter Weibull distribution analysis (Equation (5) on 16 independent samples:

(5) $$P\left(E_{b}\right)=1-e^{-{\left(\frac{E_{b}}{\alpha }\right)}^{\beta }}$$

where *E*_b_ is the Weibull breakdown strength at 63.2% of cumulative failure probability and *β* is the shape parameter that reflects data dispersion.

For large‐area film testing, 60 independent devices were analyzed using the IQR method. The dataset was divided into quartiles via linear interpolation. All statistical analyses were performed using MATLAB 2023a.

## Conflict of Interest

The authors declare no conflict of interest.

## Author Contributions

Y.L., L.F., and Z.X. conceived the idea. Y.L., Z.X., and L.F. designed the experiments. X.C. carried out a quantum chemistry calculation. L.F., Z.X., and A.D. prepared device samples for measurements under the supervision of Y.L. L.F., Z.X., Q.Z., S.L., Z.H., T.C., A.D., L.M.K., and S.W.S. carried out structural, electrical, thermal, mechanical, and optical experiments under the supervision of Y.L., Y.Y., J.Z., and T.X. H.G. and M.C. conducted charge measurements under the supervision of Y.W., T.H., and Y.Y. X.P. carried out FEM simulations with help from Z.X. under the supervision of P.L. and Z.P. Y.L., F.L., Z.X., and H.L. wrote the first version of the manuscript with help from Y.Y. All authors discussed the results and provided inputs to the manuscript.

## Supporting information



Supporting Information

## Data Availability

The data that support the findings of this study are available in the supplementary material of this article.

## References

[1] X. Qian , X. Chen , L. Zhu , Q. M. Zhang , Science 2023, 380, adg0902. 10.1126/science.adg0902 37167372

[2] H. Li , Y. Zhou , Y. Liu , L. Li , Y. Liu , Q. Wang , Chem. Soc. Rev. 2021, 50, 6369. 34100032 10.1039/d0cs00765j

[3] K. Fan , X. Li , X. Liu , X. He , Z.‐M. Dang , Adv. Mater. 2025, 37, 2417181. 10.1002/adma.202417181 40150945

[4] J. Chen , Y. Zhou , X. Huang , C. Yu , D. Han , A. Wang , Y. Zhu , K. Shi , Q. Kang , P. Li , P. Jiang , X. Qian , H. Bao , S. Li , G. Wu , X. Zhu , Q. Wang , Nature 2023, 615, 62. 36859585 10.1038/s41586-022-05671-4

[5] X. Wu , X. Chen , Q. M. Zhang , D. Q. Tan , Energy Storage Mater. 2022, 44, 29.

[6] Z. Meng , T. Zhang , C. Zhang , Y. Shang , Q. Lei , Q. Chi , Adv. Mater. 2024, 36, 2310272. 10.1002/adma.202310272 38109702

[7] H. Tran , R. Gurnani , C. Kim , G. Pilania , H.‐K. Kwon , R. P. Lively , R. Ramprasad , Nat. Rev. Mater. 2024, 9, 866.

[8] M. Yang , W. Ren , Z. Jin , E. Xu , Y. Shen , Nat. Commun. 2024, 15, 8647. 39368966 10.1038/s41467-024-52791-8 PMC11455895

[9] T. Zhang , H. Sun , C. Yin , Y. H. Jung , S. Min , Y. Zhang , C. Zhang , Q. Chen , K. J. Lee , Q. Chi , Prog. Mater. Sci. 2023, 140, 101207.

[10] M. Yang , M. Guo , E. Xu , W. Ren , D. Wang , S. Li , S. Zhang , C.‐W. Nan , Y. Shen , Nat. Nanotechnol. 2024, 19, 588. 38172431 10.1038/s41565-023-01541-w

[11] T.‐Y. Wang , J. Mao , B. Zhang , G.‐X. Zhang , Z.‐M. Dang , Nat. Rev. Electr. Eng. 2024, 1, 516.

[12] Z. Xie , L. Fan , H. Li , Z. Ran , S. Lai , X. Liu , A. Deatherage , Y. Wang , Q. Li , Y. Yin , Y. Liu , Prog. Polym. Sci. 2025, 164, 101957.

[13] X.‐J. Liu , M.‐S. Zheng , G. Chen , Z.‐M. Dang , J.‐W. Zha , Energy Environ. Sci. 2022, 15, 56.

[14] Z. Pan , L. Li , L. Wang , G. Luo , X. Xu , F. Jin , J. Dong , Y. Niu , L. Sun , C. Guo , W. Zhang , Q. Wang , H. Wang , Adv. Mater. 2023, 35, 2207580. 10.1002/adma.202207580 36333878

[15] X. Li , P. Hu , J. Jiang , J. Pan , C.‐W. Nan , Y. Shen , Adv. Mater. 2025, 37, 2411507. 10.1002/adma.202411507 39846312

[16] G. Rui , J. (.J.). Bernholc , S. Zhang , Q. Zhang , Adv. Mater. 2024, 36, 2311739. 10.1002/adma.202311739 38345782

[17] Z. Xie , K. Le , H. Li , X. Pang , T. Xu , V. Altoé , L. M. Klivansky , Y. Wang , Z. Huang , S. W. Shelton , X. Gu , P. Liu , Z. Peng , Y. Liu , Adv. Funct. Mater. 2024, 34, 2314910.

[18] X. Wu , A. Karlin , V. Beilin , G. E. Shter , G. S. Grader , Y. Ivry , S. Lin , D. Q. Tan , Adv. Mater. 2024, 36, 2401597. 10.1002/adma.202401597 38511907

[19] Q. Li , L. Chen , M. R. Gadinski , S. Zhang , G. Zhang , H. U. Li , E. Iagodkine , A. Haque , L.‐Q. Chen , T. N. Jackson , Q. Wang , Nature 2015, 523, 576. 26223625 10.1038/nature14647

[20] E. Vargo , L. Ma , H. Li , Q. Zhang , J. Kwon , K. M. Evans , X. Tang , V. L. Tovmasyan , J. Jan , A. C. Arias , H. Destaillats , I. Kuzmenko , J. Ilavsky , W.‐R. Chen , W. Heller , R. O. Ritchie , Y. Liu , T. Xu , Nature 2023, 623, 724. 37938779 10.1038/s41586-023-06660-x

[21] H. Li , E. Vargo , Z. Xie , L. Ma , P. F. Pieters , S. W. Shelton , A. P. Alivisatos , T. Xu , Y. Liu , Adv. Mater. 2024, 36, 2401954. 10.1002/adma.202401954 38669470

[22] B. V. Tawade , M. Singh , I. E. Apata , J. Veerasamy , N. Pradhan , A. Karim , J. F. Douglas , D. Raghavan , JACS Au 2023, 3, 1365. 37234129 10.1021/jacsau.3c00022 PMC10207098

[23] Z. Wang , Y. Zhao , M. Yang , H. Yan , C. Xu , B. Tian , C. Zhang , Q. Xie , Z.‐M. Dang , Adv. Energy Mater. 2025, 15, 2405411.

[24] M. Yang , H. Li , J. Wang , W. Shi , Q. Zhang , H. Xing , W. Ren , B. Sun , M. Guo , E. Xu , N. Sun , L. Zhou , Y. Xiao , M. Zhang , Z. Li , J. Pan , J. Jiang , Z. Shen , X. Li , L. Gu , C.‐W. Nan , X. Wang , Y. Shen , Nat. Energy 2024, 9, 143.

[25] X. Li , B. Liu , J. Wang , S. Li , X. Zhen , J. Zhi , J. Zou , B. Li , Z. Shen , X. Zhang , S. Zhang , C.‐W. Nan , Nat. Commun. 2024, 15, 6655. 39107376 10.1038/s41467-024-51052-y PMC11303793

[26] Z. Xie , Z. Huang , H. Li , T. Xu , H. Zhao , Y. Wang , X. Pang , Z. Cao , V. Altoé , L. M. Klivansky , Z. Wang , S. W. Shelton , S. Lai , P. Liu , C. Zhu , M. D. Connolly , C. Y. Ralston , X. Gu , Z. Peng , J. Zhang , Y. Liu , Energy Environ. Sci. 2025, 18, 620.

[27] X. Dong , B. Wan , J. W. Zha , Chem. Rev. 2024, 124, 7674. 38847509 10.1021/acs.chemrev.3c00802

[28] H. Luo , X. Zhou , C. Ellingford , Y. Zhang , S. Chen , K. Zhou , D. Zhang , C. R. Bowen , C. Wan , Chem. Soc. Rev. 2019, 48, 4424. 31270524 10.1039/c9cs00043g

[29] Y. Zhou , Y. Zhu , W. Xu , Q. Wang , Adv. Energy Mater. 2023, 13, 2203961.

[30] C. Yuan , Y. Zhou , Y. Zhu , J. Liang , S. Wang , S. Peng , Y. Li , S. Cheng , M. Yang , J. Hu , B. Zhang , R. Zeng , J. He , Q. Li , Nat. Commun. 2020, 11, 3919. 32764558 10.1038/s41467-020-17760-x PMC7411043

[31] W. E. Piers , T. Chivers , Chem. Soc. Rev. 1997, 26, 345.

[32] R. J. Mayer , N. Hampel , A. R. Ofial , Chemistry 2021, 27, 4070. 33215760 10.1002/chem.202003916 PMC7985883

[33] D. W. Stephan , Science 2016, 354, aaf7229.27940818

[34] B. Yurash , D. X. Cao , V. V. Brus , D. Leifert , M. Wang , A. Dixon , M. Seifrid , A. E. Mansour , D. Lungwitz , T. Liu , P. J. Santiago , K. R. Graham , N. Koch , G. C. Bazan , T.‐Q. Nguyen , Nat. Mater. 2019, 18, 1327. 31527809 10.1038/s41563-019-0479-0

[35] P. Pingel , M. Arvind , L. Kölln , R. Steyrleuthner , F. Kraffert , J. Behrends , S. Janietz , D. Neher , Adv. Electron. Mater. 2016, 2, 201600204.

[36] P. S. Marqués , G. Londi , B. Yurash , T.‐Q. Nguyen , S. Barlow , S. R. Marder , D. Beljonne , Chem. Sci. 2021, 12, 7012. 34123329 10.1039/d1sc01268a PMC8153436

[37] B. Yurash , D. Leifert , G. N. M. Reddy , D. X. Cao , S. Biberger , V. V. Brus , M. Seifrid , P. J. Santiago , A. Köhler , B. F. Chmelka , G. C. Bazan , T.‐Q. Nguyen , Chem. Mater. 2019, 31, 6715.

[38] E. H. Suh , S. B. Kim , H. S. Yang , J. Jang , Adv. Funct. Mater. 2022, 32, 202207413.

[39] D. J. Parks , W. E. Piers , M. Parvez , R. Atencio , M. J. Zaworotko , Organometallics 1998, 17, 1369.

[40] M. M. Hansmann , A. López‐Andarias , E. Rettenmeier , C. Egler‐Lucas , F. Rominger , A. S. K. Hashmi , C. Romero‐Nieto , Angew. Chem., Int. Ed. 2016, 55, 1196. 10.1002/anie.201508461 26663428

[41] D. Vagedes , R. Fröhlich , G. Erker , Angew. Chem., Int. Ed. 1999, 38, 3362. 10.1002/(sici)1521-3773(19991115)38:22<3362::aid-anie3362>3.0.co;2-n 10602195

[42] J. G. Simmons , M. C. Tam , Phys. Rev. B 1973, 7, 3706.

[43] H. Li , B. S. Chang , H. Kim , Z. Xie , A. Lainé , L. Ma , T. Xu , C. Yang , J. Kwon , S. W. Shelton , L. M. Klivansky , V. Altoé , B. Gao , A. M. Schwartzberg , Z. Peng , R. O. Ritchie , T. Xu , M. Salmeron , R. Ruiz , K. B. Sharpless , P. Wu , Y. Liu , Joule 2023, 7, 95. 37034575 10.1016/j.joule.2022.12.010 PMC10078921

[44] J.‐Y. Pei , L.‐J. Yin , S.‐L. Zhong , Z.‐M. Dang , Adv. Mater. 2023, 35, 2203623. 10.1002/adma.202203623 35924412

[45] J.‐Y. Pei , J. Zhu , L.‐J. Yin , Y. Zhao , M. Yang , S.‐L. Zhong , Q.‐K. Feng , Z.‐M. Dang , Adv. Funct. Mater. 2024, 34, 2316869.

[46] I. B. Sivaev , V. I. Bregadze , Coord. Chem. Rev. 2014, 270, 75.

[47] J. N. Bentley , S. A. Elgadi , J. R. Gaffen , P. Demay‐Drouhard , T. Baumgartner , C. B. Caputo , Organometallics 2020, 39, 3645.

[48] J. R. Gaffen , J. N. Bentley , L. C. Torres , C. Chu , T. Baumgartner , C. B. Caputo , Chem 2019, 5, 1567.

[49] F. Neese , Wiley Interdiscip. Rev.‐Comput. Mol. Sci. 2012, 2, 73.

[50] S. Grimme , S. Ehrlich , L. Goerigk , J. Comput. Chem. 2011, 32, 1456. 21370243 10.1002/jcc.21759

[51] T. Lu , F. W. Chen , J. Theor. Comput. Chem. 2012, 11, 163.

[52] T. Lu , F. Chen , J. Comput. Chem. 2012, 33, 580. 22162017 10.1002/jcc.22885

[53] W. Humphrey , A. Dalke , K. Schulten , J. Mol. Graph. Model. 1996, 14, 33. 10.1016/0263-7855(96)00018-5 8744570

[54] Y. Wang , J. Wu , L. Fan , M. Chen , Y. Yin , IEEE Trans. Dielectr. Electr. Insul. 2024, 31, 1685.

