# Supplementary material for: Lewis Acid‐Activated Charge Trapping in Dielectric Polymers for Superior High‐Temperature Electrostatic Energy Storage
Source: Adv Sci (Weinh). 2025 Nov 19;13(7):e17934. doi: 10.1002/advs.202517934 (PMC12866723; doi:10.1002/advs.202517934)
Supplement: Supplementary file 1 — Supporting Information [file ADVS-13-e17934-s001.pdf]

Supporting Information

Lewis Acid-Activated Charge Trapping in Dielectric Polymers for Superior High-Temperature Electrostatic Energy Storage

Lu Fan, Zongliang Xie, Xi Chen, Qingsong Zhang, Yalin Wang, He Li, Xi Pang, Tiffany Chen, Shiqi Lai, Zhiyuan Huang, Ashlin M. Deatherage, Hanjiang Gu, Meng Chen, Tao Han, Liana M. Klivansky, Steve W. Shelton, Peng Liu, Zongren Peng, Ting Xu, Jian Zhang, Yi Yin and Yi Liu**

L. Fan, Z. Xie, Q. Zhang, H. Li, S. Lai, Z. Huang, A. M. Deatherage, L. M. Klivansky, S. W. Shelton, J. Zhang, Y. Liu
The Molecular Foundry, Lawrence Berkeley National Laboratory, Berkeley, CA 94720, USA
E-mail: yliu@lbl.gov

L. Fan, Y. Wang, H. Gu, M. Chen, Y. Yin
School of Electrical Engineering, Shanghai Jiao Tong University, Shanghai 200240, China
E-mail: yiny@sjtu.edu.cn

Z. Xie, H. Li, T. Chen, T. Xu, Y. Liu
Materials Sciences Division, Lawrence Berkeley National Laboratory, Berkeley, California 94720, USA

X. Chen
School of Materials Science and Engineering, Nanyang Technological University, Singapore 639798, Singapore

X. Pang, P. Liu, Z. Peng
State Key Laboratory of Electrical Insulation and Power Equipment, Xi'an Jiaotong University, Xi'an, Shaanxi 710049, China.

T. Chen, S. Lai, A. M. Deatherage, T. Xu
Department of Materials Science and Engineering, University of California, Berkeley, Berkeley, California 94720, USA

T. Han
School of Automation and Intelligent Sensing, Shanghai Jiao Tong University, Shanghai 200240, China

T. Xu
Department of Materials Science and Engineering, University of California, Berkeley, CA 94720, USA

Note S1. General information, synthesis, film preparation, characterization method.

Materials: All chemicals were purchased from commercial sources and used directly without purification unless otherwise noted. Polyetherimide (Ultem 1000, PEI) pellets and Fluorene Polyester (FPE) powders were purchased from PolyK Technologies, LLC. Soluble polyimide (Matrimid 5218, sPI) powders and polyimide with -CF_3 groups (KPI-MX300F, FPI) powders were purchased from Kawamura. Tris(pentafluorophenyl)boron (BCF) and triphenylboron (TPB) were purchased from TCI. 2-Phenylisoindole-1,3-dione (PID) was purchased from Aaron Chemicals and purified before use. 5-Phenoxyisobenzofuran-1,3-dione was purchased from AmBeed and purified before use. All solvents were purchased from Sigma-Aldrich.

General characterization method: Solution ^{1}H, ^{19}F and ^{13}C NMR spectra of PEI, BCF and PEI-BCF (molar ratio 1:1) were recorded on a Bruker Avance II 500 spectrometer. UV-vis absorption spectra of PEI, BCF and PEI-BCF (molar ratio 1:1) were obtained on an Agilent Cary 5000 UV-vis-NIR spectrometer. The infrared spectra were recorded in a Nicolet iS50 FTIR from ThermoFisher collected over the mid-IR region using a KBr beamsplitter and a built-in diamond crystal attenuated total reflectance (ATR). X-ray photoelectron spectroscopy (XPS) was performed on a Thermo Scientific™ K-AlphaPlus™ instrument equipped with monochromatic Al Kα radiation (1486.7 eV) as the excitation source. The X-ray analysis area for measurement was set at 200 × 400 µm (ellipse shape) and a flood gun was used for charge compensation. The pass energy was 200 eV for the wide (survey) spectra and 50 eV for the high-resolution regions (narrow spectra). The base pressure of the analysis chamber was less than ~1 × 10^{-9} mbar. The analysis chamber pressure was at 1 × 10^{-7} mbar during data acquisition.

Synthesis of PPPI: To synthesize 4-phenoxy-N-phenyl-phthalimide (PPPI), the commercially available 5-Phenoxyisobenzofuran-1,3-dione (1.0 mmol, 240 mg, 1.0 eq) was mixed with aniline (1.0 mmol, 93 mg, 1.0 eq) in a 10 mL microwave vial and dissolved in dry NMP (2 mL) in a nitrogen atmosphere. The vial was heated at 185 °C for eight hours in an oven. The black reaction mixture was quenched with 5 mL of water. The filter cake was washed twice by water, EtOH, and hexanes, respectively, to give a white powder (310 mg, 93%), which was recrystallized to give the final product. ^{1}H NMR (500 MHz, CD_2Cl_2, 298 K) δ 7.88 (dd, *J* = 8.0, 0.5 Hz, 1H), 7.53~7.38 (m, 8H), 7.36 (dd, *J* = 8.0, 2 Hz, 1H), 7.29 (tt, *J* = 7.0, 1.5 Hz, 1H), 7.16~7.13 (m, 2H). ^{13}C NMR (126 MHz, CD_2Cl_2, 298 K) δ 167.09, 167.02, 164.14, 155.28, 134.69, 132.32, 130.78, 129.38, 128.45, 127.10, 125.93, 125.80, 125.60, 123.30, 120.84, 112.16, see Supplementary Figs. 1 and 2.

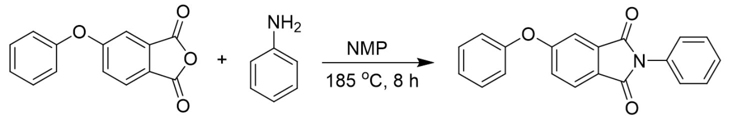

Polymeric film preparation: The PEI-BCF and PEI-TPB solutions were processed in a glovebox, wherein PEI pellets were magnetically stirred in NMP to yield a clear solution (40 mg ml^{-1}). A predetermined quantity of BCF or TPB was added to the solution and the resulting mixture was subsequently shaken for 5 min. The solution was immediately cast on clean glass slides and kept in a vacuum oven at 95 °C for 12 h, followed by annealing at 200 °C for 12 h to ensure the removal of residual solvent. FPE-BCF solution and sPI-BCF composites were processed using the same process as PEI. For FPI, DMF was chosen as the solvent while the rest of the steps were the same as above. The thickness of resulting films was kept at 12 ± 2 µm unless noted otherwise.

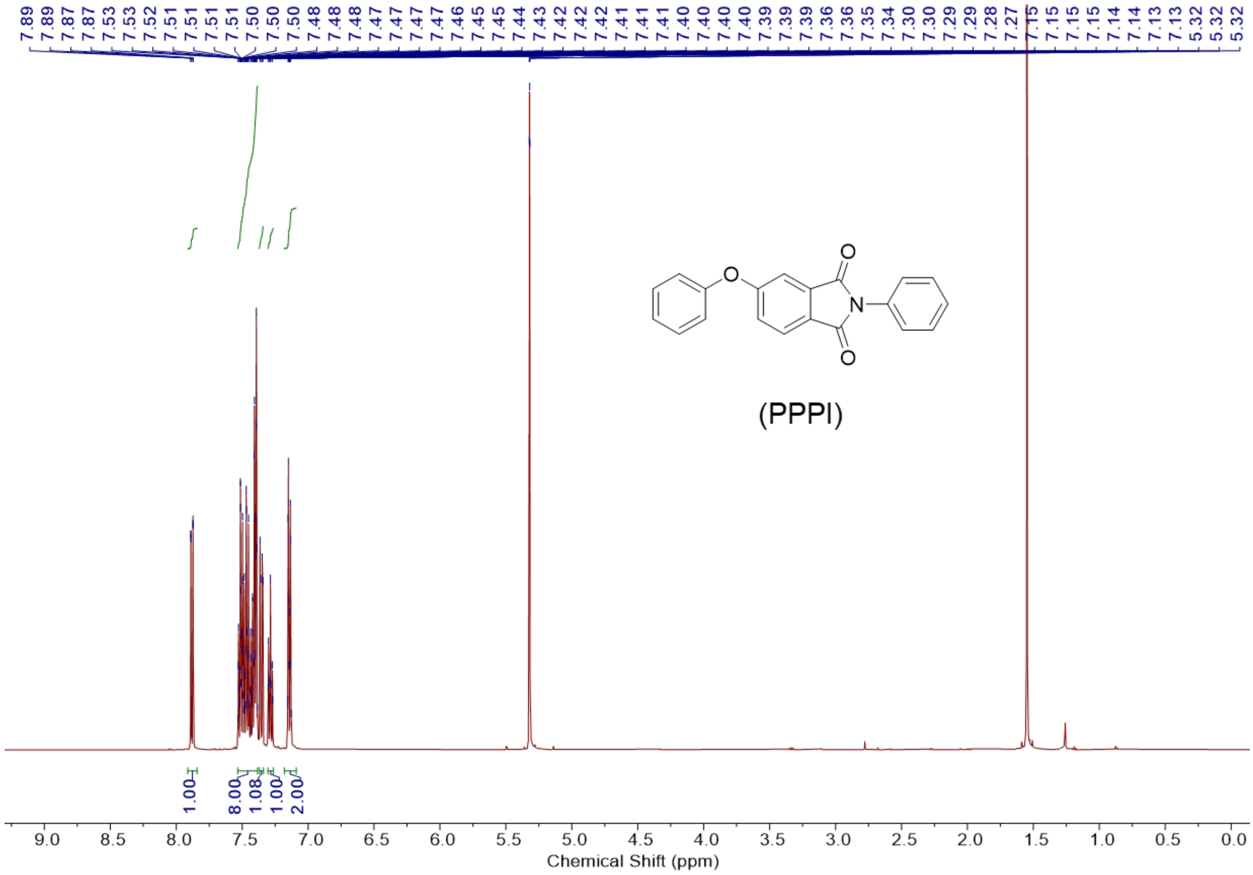

Figure S1. ^{1}H NMR (500 MHz, CD_2Cl_2, 298 K) spectrum of PPPI.

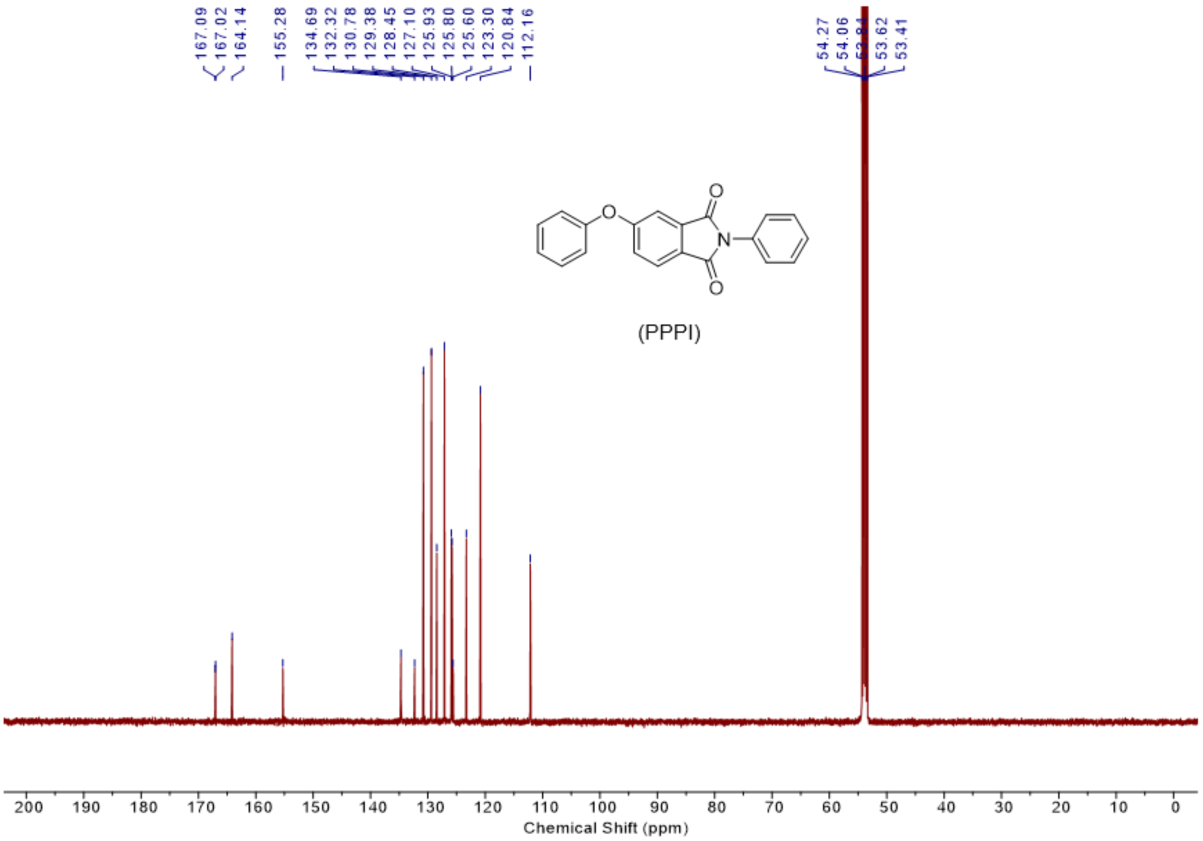

Figure S2. ^{13}C NMR (126 MHz, CD_2Cl_2, 298 K) spectrum of PPPI.

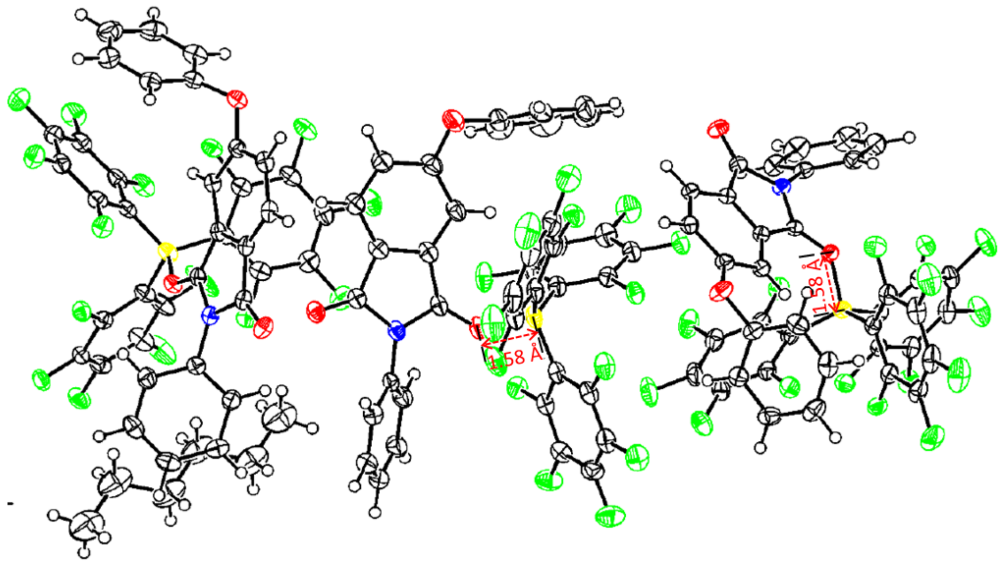

Figure S3. An asymmetric unit in the single crystal structure of PPPI-BCF. The PPPI-BCF unit is drawn in wireframe form using thermal ellipsoids drawn to the 50% probability level, where C atoms are black ellipsoid, H atoms are white ellipsoid, O atoms are red ellipsoid, B atoms are yellow ellipsoid, F atoms are green ellipsoid, and N atoms are blue ellipsoid.

Table S1. Crystallographic parameters for PPPI-BCF.

	**PPPI-BCF**
Empirical formula	$BC_{33}Cl_2F_{15}NO_2H_{11}$
Formula weight	820.14
Temperature/K	100
Crystal system	monoclinic
Space group	$P2_1/c$
a/Å	15.9620(17)
b/Å	12.7361(13)
c/Å	15.9682(17)
α/°	90
β/°	107.174(4)
γ/°	90
Volume/Å^3	3101.5(6)
Z	4
ρ_{calc}g/cm^3	1.756
μ/mm^{-1}	0.357
F(000)	1624.0
Crystal size/mm^3	0.2 × 0.08 × 0.06
Radiation	synchrotron (λ = 0.7288)
2Θ range for data collection/°	4.272 to 58.24
Index ranges	-21 ≤ h ≤ 21, -16 ≤ k ≤ 16, -21 ≤ l ≤ 21
Reflections collected	113291
Independent reflections	7703 [R_{int} = 0.0567, R_{sigma} = 0.0284]
Data/restraints/parameters	7703/0/488
Goodness-of-fit on F^2	1.070
Final R indexes [I>=2σ (I)]	R_1 = 0.0335, wR_2 = 0.0854
Final R indexes [all data]	R_1 = 0.0360, wR_2 = 0.0873
Largest diff. peak/hole / e Å^{-3}	0.48/-0.39

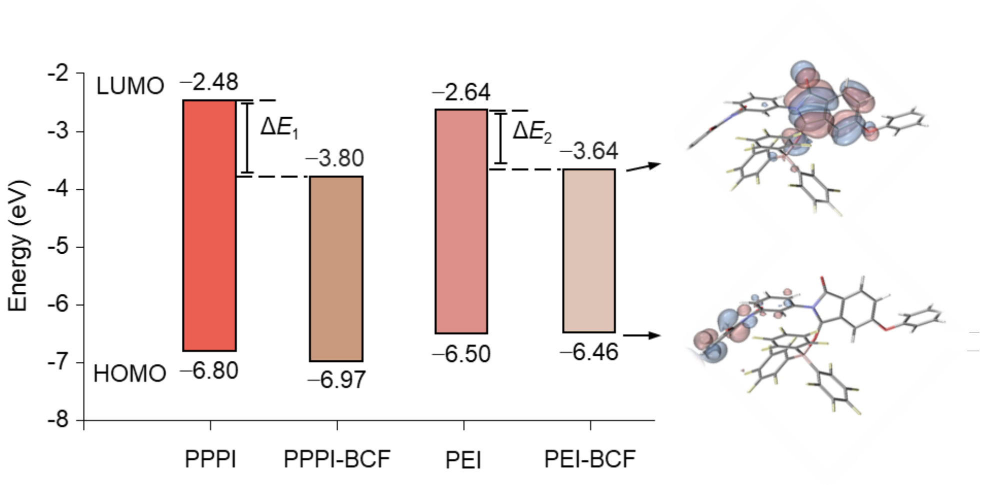

Figure S4. Comparison of frontier energy levels of the molecular analog PPPI, a dimeric segment of PEI and their respective BCF complexes. The LUMO and HOMO energy levels were computed based on energy minimized structures except PPPI–BCF which was based on its crystal structure. A dimeric segment of PEI was used to represent PEI in all the simulations. Also plotted were frontier orbitals (isoelectric value: ±0.02) of the simulated PEI-BCF complex.

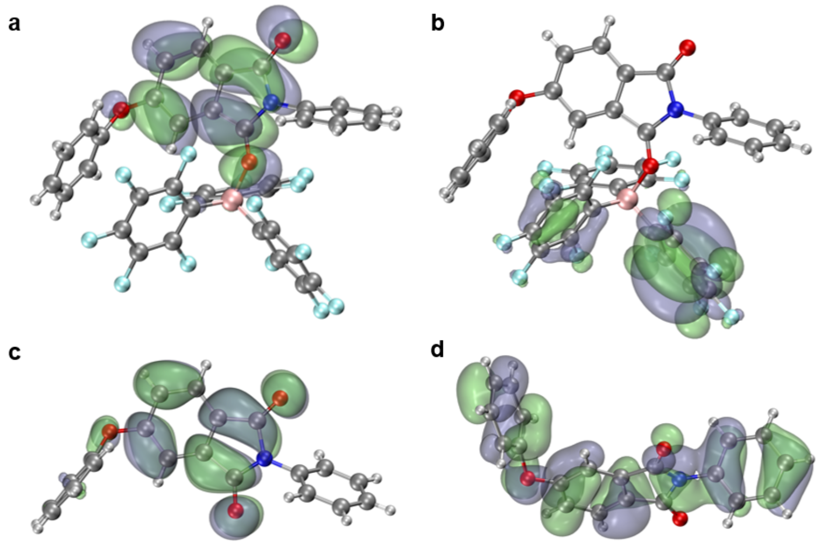

Figure S5. The LUMO and HOMO orbitals (isovalue ±0.02) of (a), (b) the PPPI-BCF complex (LUMO level: -3.80 eV, HOMO level: -6.97 eV) and (c), (d) the PPPI monomer (LUMO level: -2.48 eV, HOMO level: -6.80 eV).

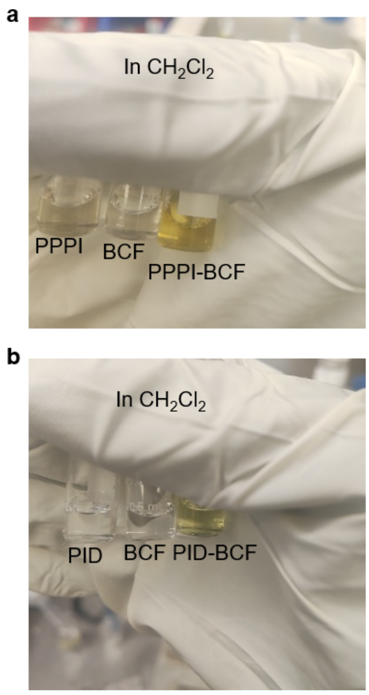

Figure S6. Optical images of (a) PPPI, BCF and PPPI-BCF (molar ratio 1:1) and (b) PID, BCF and PID-BCF (molar ratio 1:1) in CH_2Cl_2 solutions.

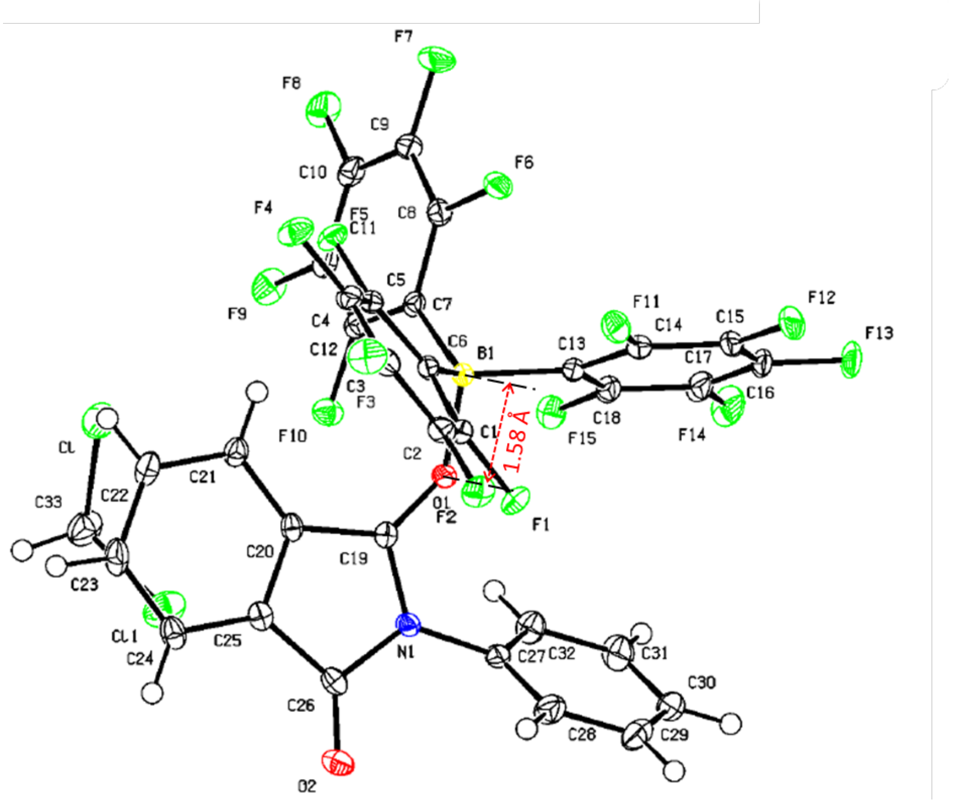

Figure S7. An asymmetric unit in the single crystal structure of PID-BCF. The PID-BCF unit is drawn in wireframe form using thermal ellipsoids drawn to the 50% probability level. C atoms are black ellipsoid, H atoms are white ellipsoid, O atoms are red ellipsoid, B atoms are yellow ellipsoid, F atoms are green ellipsoid, and N atoms are blue ellipsoid.

Table S2. Crystallographic parameters for PID-BCF.

	**PID-BCF**
Empirical formula	$C_{117}H_{46}B_3F_{45}N_3O_9$
Formula weight	2525.00
Temperature/K	100
Crystal system	triclinic
Space group	P-1
a/Å	11.9099(14)
b/Å	20.305(2)
c/Å	22.853(3)
α/°	71.503(4)
β/°	79.785(4)
γ/°	84.578(4)
Volume/Å^3	5153.6(10)
Z	2
ρ_{calc}g/cm^3	1.627
μ/mm^{-1}	0.167
F(000)	2522.0
Crystal size/mm^3	0.12 × 0.07 × 0.055
Radiation	synchrotron (λ = 0.7288)
2Θ range for data collection/°	3.334 to 58.338
Index ranges	-15 ≤ h ≤ 15, -27 ≤ k ≤ 27, -30 ≤ l ≤ 30
Reflections collected	136452
Independent reflections	25693 [R_{int} = 0.0665, R_{sigma} = 0.0485]
Data/restraints/parameters	25693/0/1595
Goodness-of-fit on F^2	1.041
Final R indexes [I>=2σ (I)]	R_1 = 0.0515, wR_2 = 0.1417
Final R indexes [all data]	R_1 = 0.0681, wR_2 = 0.1532
Largest diff. peak/hole / e Å^{-3}	0.98/-0.41

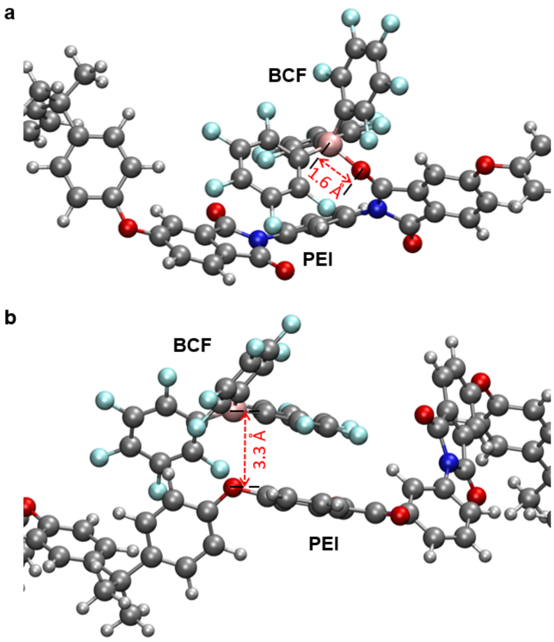

Figure S8. Energy-optimized structures of complexes formed between BCF and a dimeric segment of PEI based on interactions between boron and (a) carboxyl oxygen atom (B•••O distance 1.6 Å) and (b) etheric oxygen atom (B•••O distance 3.3 Å) in PEI chains. The carbon atoms are dark gray, hydrogen atoms are light gray, oxygen atom are red, nitrogen atoms are blue, boron atoms are pink, and fluorine atoms are light blue.

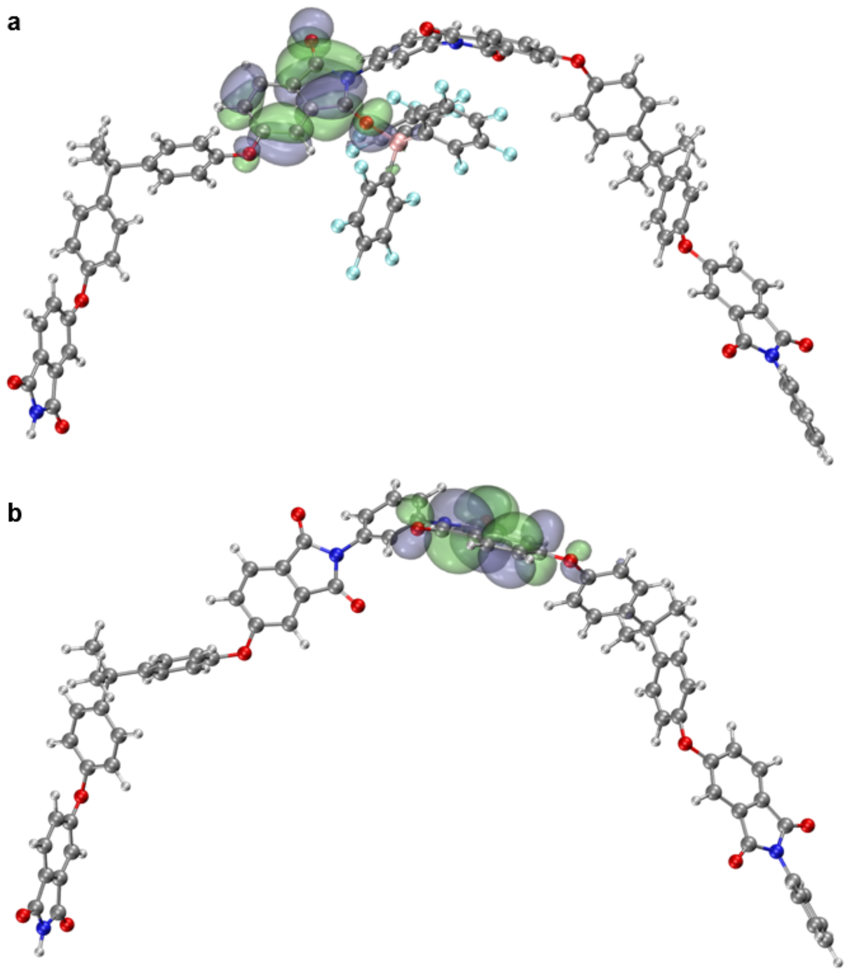

Figure S9. LUMO orbitals (isovalue ±0.02) of (a) the dimeric PEI-BCF complexes (b) the dimeric PEI. The B•••O coordination localized the LUMO orbital near the coordination site.

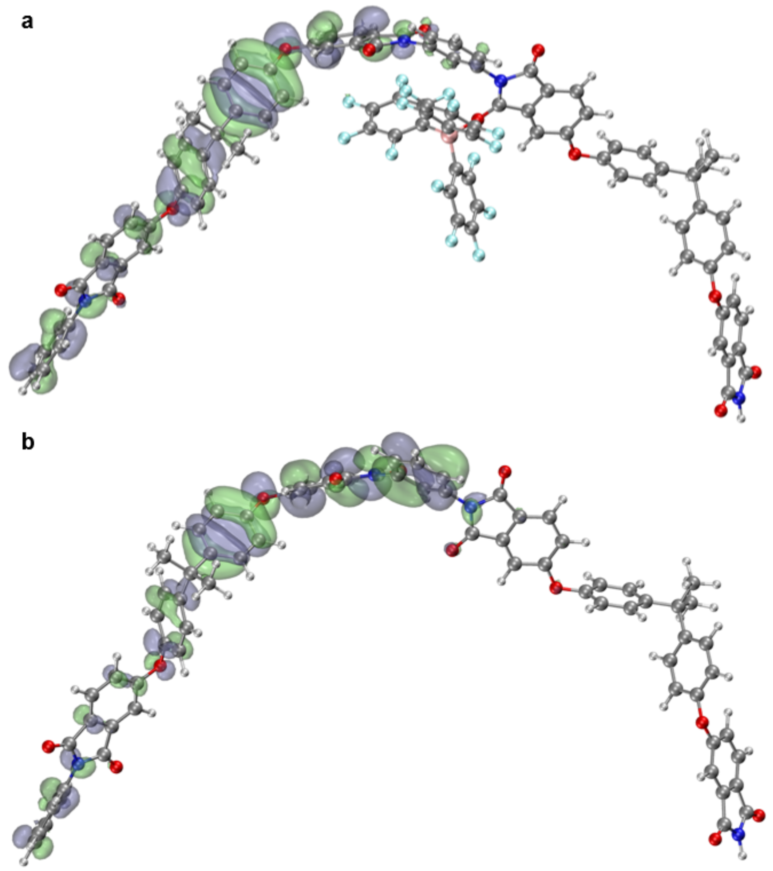

Figure S10. HOMO orbitals (isovalue ±0.02) of (a) the dimeric PEI-BCF complexes and (b) the dimeric PEI.

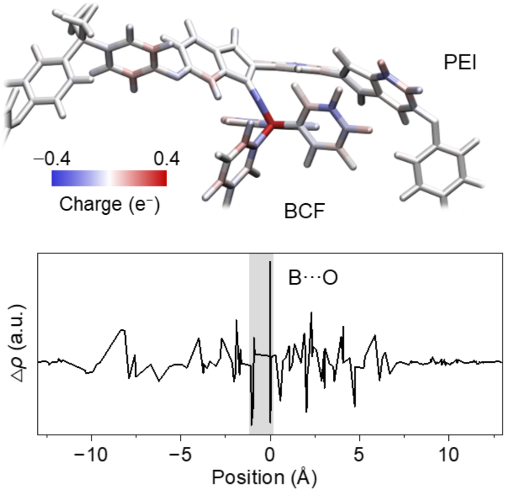

Figure S11. Calculated charge transfer of each atom in the complex between BCF and a dimeric PEI segment. Red color indicated electron gain and blue color indicated electron loss. The bottom plot showed charge transfer of each atom along the X-axis, wherein Boron atom was set at coordinate zero.

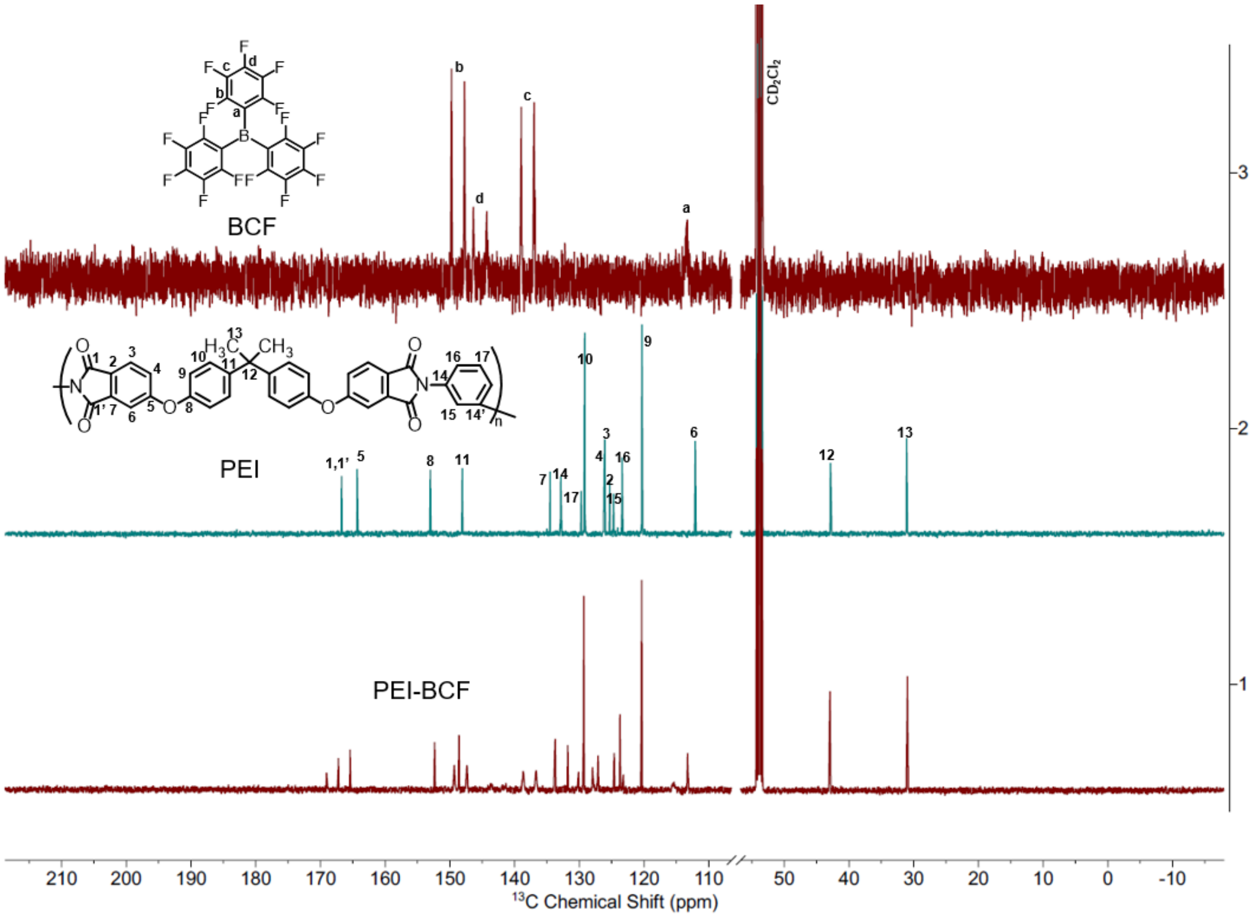

Figure S12. ^{13}C NMR spectroscopy of BCF (top), PEI (center) and PEI-BCF complex (bottom). BCF, ^{13}C NMR (126 MHz, CD_2Cl_2, 298 K): δ 150.3–147.6 (m), 147.1–144.1 (m), 139.6–136.7 (m), 113.9–113.3 (m). PEI, ^{13}C NMR (126 MHz, CD_2Cl_2, 298 K): δ 166.74, 166.71, 164.32, 153.02, 148.08, 134.51, 132.87, 129.70, 129.15, 126.16, 126.05, 125.30, 124.68, 124.06, 123.39, 120.33, 112.06, 42.86, 31.10. PEI-BCF complex, ^{13}C NMR (126 MHz, CD_2Cl_2, 298 K): δ 169.03 167.22, 165.42, 152.36, 149.31, 148.58, 147.35, 143.58, 138.67, 136.68, 133.72, 131.78, 130.12, 129.31, 127.95, 127.10, 124.60, 123.73, 123.19, 120.37, 115.53, 113.27, 42.94, 30.99.

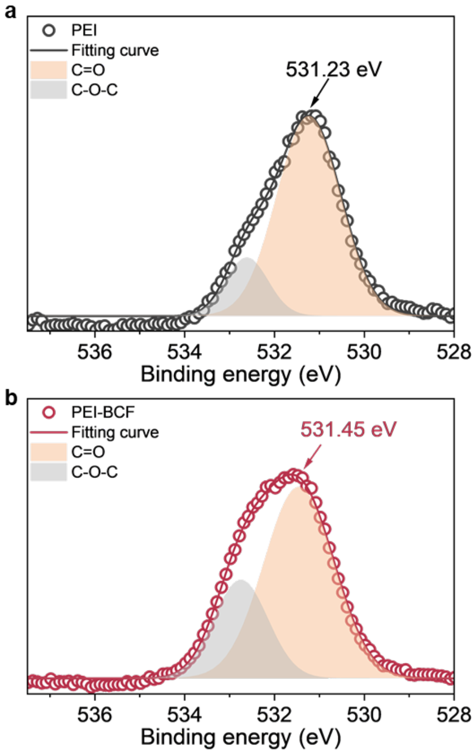

Figure S13. X-ray photoelectron spectroscopy (XPS) of O1s peaks in (a) PEI and (b) PEI-BCF films.

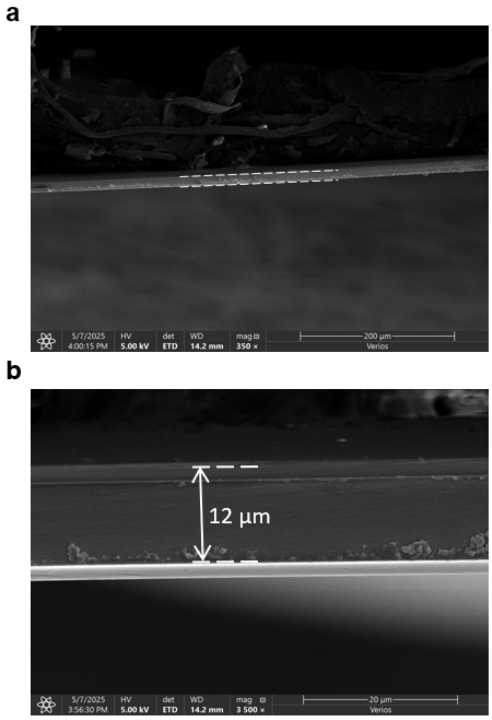

Figure S14. Scanning electron microscope (SEM) images of PEI-$BCF_{0.5}$ film at magnifications of (a) 350 and (b) 3500. The scalebars are 200 µm and 20 µm, respectively.

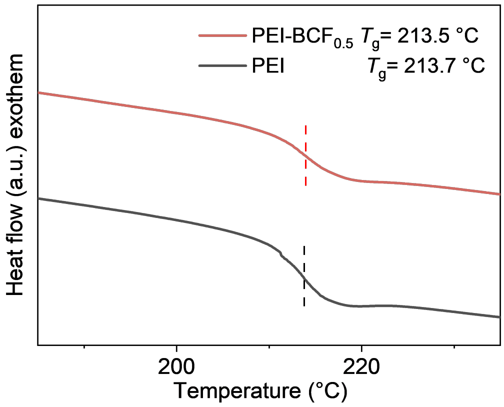

Figure S15. DSC curves of the PEI and PEI-$BCF_{0.5}$ films.

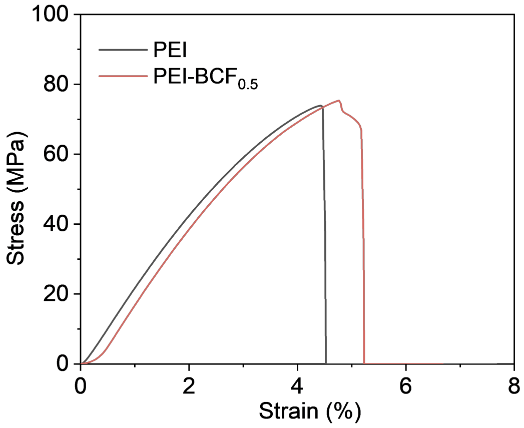

Figure S16. DMA curves of the PEI (Young's modulus is 2.05 GPa) and PEI-$BCF_{0.5}$ films (Young's modulus is 2.2 GPa).

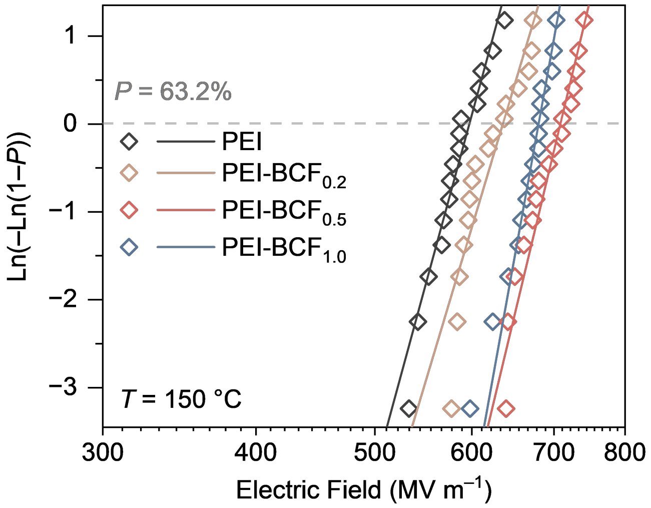

Figure S17. Weibull breakdown plots of PEI and PEI-BCF$_n$ films at 150 °C.

Table S3. Weibull breakdown parameters of PEI and PEI-BCF$_n$ films at 150 and 200 °C.

Materials	150 °C		200 °C	
	E_b (MV m^{-1})	β	E_b (MV m^{-1})	β
PEI	597	22	525	20
PEI-BCF$_{0.2}$	636	20	602	13
PEI-BCF$_{0.5}$	708	25	687	29
PEI-BCF$_1$	680	33	666	19

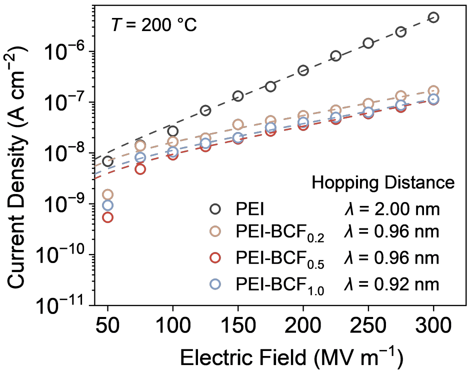

Figure S18. *J-E* curves of PEI and PEI-BCF$_n$ films at 200 °C.

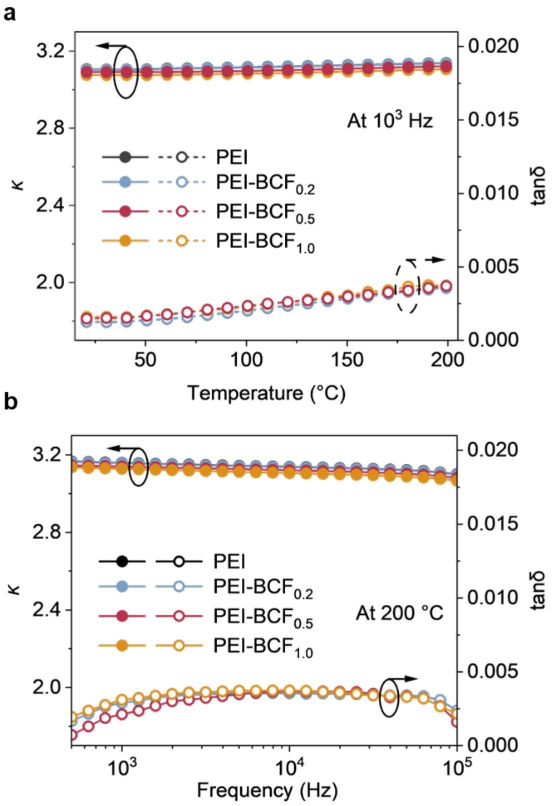

Figure S19. (a) Temperature-dependent (at 1000 Hz) and (b) Frequency-dependent (at 200 °C) dielectric spectra of pure PEI and PEI-BCF$_n$ films.

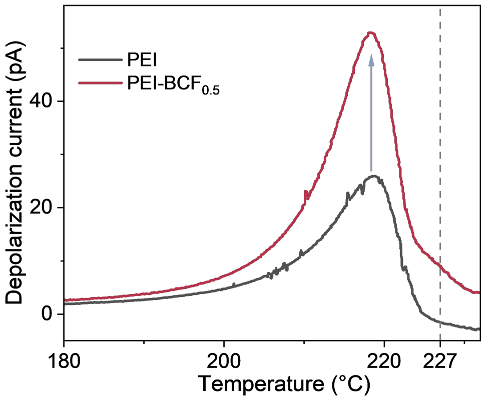

Figure S20. TSDC curves of the PEI and PEI-BCF$_{0.5}$ films.

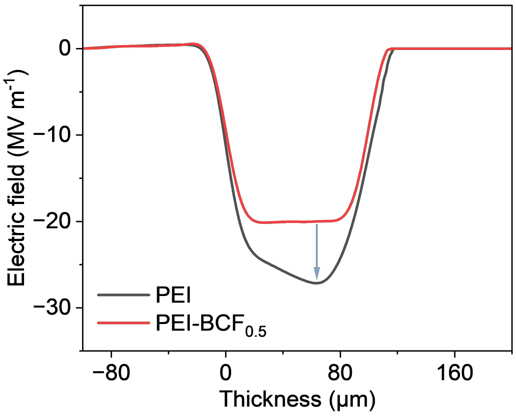

Figure S21. Electric field distribution of the PEI and PEI-BCF$_{0.5}$ films under applied electric field of 20 MV m^{-1}, calculated through Poisson's equation with space charge profile measured after keeping the film at 100 °C and 20 MV m^{-1} for 10 min.

Note S2. Bipolar charge transport dielectric breakdown (BCT-DB) simulation method.

Space charge simulation: We employed a modified two-dimensional bipolar charge transport-dielectric breakdown (2D BCT-DB) model to simulate the electric tree propagation. The bipolar carrier transport model concerns the main processes such as charge injection, migration, trapping and de-trapping, recombination and extraction.

Charge transport can be described by the current continuity equation, the Poisson equation and the transport equation as follows:

$$\begin{cases} \dfrac{\partial n_a(x,t)}{\partial t} + \dfrac{\partial f_a(x,t)}{\partial x} = S_a(x,t) \\ \dfrac{\partial E(x,t)}{\partial x} = \dfrac{\rho_{\text{all}}(x,t)}{\varepsilon_0 \varepsilon_r} \\ f_a(x,t) = \mu_a(x,t) n_a(x,t) E(x,t) - eD_f \dfrac{dn}{dx} \end{cases} \tag{S1}$$

where subscript a represents the types of carriers, including free electrons (*eμ*), free holes (*hμ*), trapped electrons (*et*) and trapped holes (*ht*); n_a is the carrier concentration, C·m^{-3}; f_a is the carrier flux density, A·m^{-2}; t is the time, s; x is the coordinate, m; ρ_{all} is the total charge density, C·m^{-3}; μ_a is the mobility of carriers, m^2·V^{-1}·s^{-1}; D_f is the diffusion coefficients, m^2·s^{-1}.

The specific expression describing the processes of trapping, de-trapping and recombination of different types of carriers is as follows:

$$\begin{cases} S_{\text{e}\mu} = -S_1 \cdot n_{\text{e}\mu} \cdot n_{\text{ht}} - S_3 \cdot n_{\text{e}\mu} \cdot n_{\text{h}\mu} - B_e \cdot n_{\text{e}\mu} \cdot (1 - \dfrac{n_{\text{et}}}{N_{\text{eto}}}) + D_e \cdot n_{\text{et}} \\ S_{\text{et}} = -S_2 \cdot n_{\text{et}} \cdot n_{\text{h}\mu} - S_0 \cdot n_{\text{et}} \cdot n_{\text{ht}} + B_e \cdot n_{\text{e}\mu} \cdot (1 - \dfrac{n_{\text{et}}}{N_{\text{eto}}}) - D_e \cdot n_{\text{et}} \\ S_{\text{h}\mu} = -S_2 \cdot n_{\text{et}} \cdot n_{\text{h}\mu} - S_3 \cdot n_{\text{e}\mu} \cdot n_{\text{h}\mu} - B_{\hbar} \cdot n_{\text{h}\mu} \cdot (1 - \dfrac{n_{\text{ht}}}{N_{\text{hto}}}) + D_{\hbar} \cdot n_{\text{ht}} \\ S_{\text{ht}} = -S_1 \cdot n_{\text{et}} \cdot n_{\text{h}\mu} - S_0 \cdot n_{\text{et}} \cdot n_{\text{ht}} + B_{\hbar} \cdot n_{\text{h}\mu} \cdot (1 - \dfrac{n_{\text{ht}}}{N_{\text{hto}}}) - D_{\hbar} \cdot n_{\text{ht}} \end{cases} \tag{S2}$$

where S_0 is the recombination coefficient of trapped electron and trapped hole, m^3·C^{-1}·s^{-1}; S_1 is the recombination coefficient of free electron and trapped hole, m^3·C^{-1}·s^{-1}; S_2 is the recombination coefficient of trapped electron and free hole, m^3·C^{-1}·s^{-1}; S_3 is the recombination coefficient of free electron and free hole, m^3·C^{-1}·s^{-1}; B_e and B_h are the trapping coefficients of electron and hole traps, respectively, s^{-1}; N_{eto} and N_{hto} are the concentrations of electron traps and hole traps, respectively, C·m^{-3}; D_e and D_h are the de-trapping coefficients of electron and hole, respectively, s^{-1}.

The charge injection from electrodes is typically described by the Schottky law as follows:

$$\begin{cases} j_{\text{ei}}(0,t) = AT^2\, exp(\dfrac{-e\omega_{\text{ei}}}{kT})\, exp(\dfrac{e}{kT}\sqrt{\dfrac{e|E(0,t)|}{4\pi\varepsilon_0\varepsilon_r}}) \\ j_{\text{hi}}(D,t) = AT^2\, exp(\dfrac{-e\omega_{\text{hi}}}{kT})\, exp(\dfrac{e}{kT}\sqrt{\dfrac{e|E(D,t)|}{4\pi\varepsilon_0\varepsilon_r}}) \end{cases} \tag{S3}$$

where $j_{ei}(0,t)$ and $j_{hi}(D,t)$ are the injection current densities at cathode (x=0) and anode (x=D), respectively, A·m^{-2}; ω_{hi} and ω_{ei} are the Schottky injection barriers for holes and electrons, respectively, eV; A=1.2×10^6 A·m^{-1}·K^{-2}, is the Richardson constant; k=1.38×10^{-23} J·K^{-1}, is the Boltzmann constant.

During the transport process, hopping conduction between carriers in shallow traps is considered as follows:

$$\mu(x,t) = \frac{2v\lambda}{E(x,t)} exp(\frac{-e\omega_{\mu}}{kT})\, sin\hbar(\frac{eE(x,t)\lambda}{2kT}) \tag{S4}$$

where $\mu(x,t)$ is the carrier mobility considering hopping conduction, m^2·V^{-1}·s^{-1}; λ is the hopping distance, nm; ω_μ is the hopping barrier height, eV; v is the attempt-to-escape frequency, which is set to 9.85×10^{12} Hz.

The free charges can be trapped in deep traps with the trapping coefficients, which are described as follows:

$$B = \frac{\mu_a \cdot N_t \cdot e}{\varepsilon_0 \cdot \varepsilon_r} \tag{S5}$$

where μ_a is the mobility of charges, m^2·V^{-1}·s^{-1}; N_t is the trap density, m^{-3}; e=1.6×10^{-19} C, is the electronic charge.

Trapped charges can escape from deep traps by overcoming a potential barrier and the de-trapping coefficient is as follows:

$$D = \nu \cdot exp(\frac{-\Delta Utr}{k_B T}) \quad \text{(S6)}$$

where ΔU_{tr} is the de-trapping barrier, eV, which is obtained from IRC results.

The extraction of carriers at the electrode is assumed to be as normal conduction process with an extraction barrier as follows:

$$j_{\mathrm{e,h}}(x,t) = n_{\mathrm{e\mu,h\mu}} \mu_{e,h} E(x,t) \quad \text{(S7)}$$

where j_e and j_h are the extraction current density of electrons and holes, respectively, A·m^{-2}; $n_{e\mu}$ and $n_{h\mu}$ are the charge density of mobile electrons and holes, respectively; μ_e and μ_h are the mobilities of electrons and holes, respectively.

The PEI polymer matrix is set as a cuboid with length=l, width=d and height=h and particle is set as a sphere with radius=r in the 2-D model. The density of BCF and PEI are ρ_{BCF} and ρ_{PEI}, respectively. The doping ratio of PEI composites is z in mass fraction. The number of doped particles can be calculated as follows:

$$N_{\mathrm{particle}} = \frac{3}{2} \cdot \frac{ld}{1 + (\frac{1}{z} - 1) \cdot \frac{\rho_{\mathrm{Particle}}}{\rho_{\mathrm{PEI}}}} \cdot \frac{1}{\pi r^2} \quad \text{(S8)}$$

Model 0 represents the filler-free PEI samples. Model 1 represents PEI-BCF$_{0.5}$. Based on the DFT simulation results shown in Figure S11, Supporting Information, the diameter of the influence region of BCF in PEI is set to be 1.5 nm. The free charges would be blocked by the injection barrier (1.49 eV) at the interface zone in Model 1. The trapping coefficients and deep trap depth are shown in Table S4, Supporting Information.

Table S4. Parameters for bipolar carrier transport model simulation.

	**Parameter**	**PEI**	**PEI-BCF$_{0.5}$**
Injection	Injection barrier for matrix	2.3 eV	2.3 eV
	Injection barrier for interface zone	/	2 eV
Transport	Hopping barrier height in matrix	1.0 eV	1.0 eV
	Hopping distance in matrix	2 nm	2 nm
	Hopping barrier height at the interface zone	/	1.49 eV
	Hopping distance in the interface zone	/	0.96 nm
Trapping & De-trapping	Trapping coefficient in the matrix	0.07 s^{-1}	0.07 s^{-1}
	Trapping coefficient at the interface zone	/	0.1 s^{-1}
	Deep trap depth at the interface zone	/	1.49 eV
Recombination	S_0, S_1, S_2	5×10^3	5×10^3
Basic	Temperature T	200 °C	200 °C
	Electric field E	500 MV·m^{-1}	500 MV·m^{-1}

Note S3. Phase-Field Model for Electrical-Thermal-Mechanical Breakdown:

The phase-field model can simulate the breakdown phase considering time and space and a phase-field variable η ($\mathbf{r}$, t) related with time (t) and space ($\mathbf{r}$) can describe the breakdown phase. η ($\mathbf{r}$, t) =1 represents the breakdown phase, η ($\mathbf{r}$, t) =0 represents the non-breakdown phase and 0< η ($\mathbf{r}$, t) <1 represents the the interface area. The phase-field model considering the effect of electric stress, thermal stress and mechanical stress was established to calculate breakdown phase, which can be parameterized by dielectric constant, electrical conductivity and Young' s modulus, respectively. The parameters such as dielectric constant, electrical conductivity and Young' s modulus for PEI phase, PEI-BCF phase and breakdown phase are different. The total free energy in phase-field model is shown below:

$$F = \int_V [f_{\text{sep}}(\eta(\boldsymbol{r})) + f_{\text{grad}}(\eta(\boldsymbol{r})) + f_{\text{elec}}(\boldsymbol{r}) + f_{\text{joule}}(\boldsymbol{r}) + f_{\text{strain}}(\boldsymbol{r})]dV \quad \text{(S9)}$$

where f_{sep} is the free energy density of mixing that drives the phase separation; f_{grad} is the gradient energy density; f_{elec} is the electric energy density; f_{joule} is the Joule heat energy density; f_{strain} is the strain energy density.

The phase separation energy can be described by double-well function, which is shown below:

$$f_{\text{sep}}(\eta(\boldsymbol{r})) = \alpha\eta^2(1-\eta)^2 \quad \text{(S10)}$$

where α is the energy barrier of phase separation.

The gradient energy density is shown below:

$$f_{\text{grad}} = -\frac{1}{2}\gamma|\nabla\eta(\boldsymbol{r})|^2 \quad \text{(S11)}$$

where γ is the gradient energy coefficient.

The electric energy density is described as follows:

$$f_{\text{elec}} = -\frac{1}{2}\varepsilon_0\varepsilon_{\text{ij}}(\boldsymbol{r})E_i(\boldsymbol{r})E_j(\boldsymbol{r}) \quad \text{(S12)}$$

where ε_{ij}($\mathbf{r}$) is the relative dielectric constant tensor related to space; E_{i} and E_{j} is respectively the component of the total electric field.

The Joule heat energy density can be written as follows:

$$f_{\text{joule}} = -\sigma_{\text{ij}}(\boldsymbol{r})E_i(\boldsymbol{r})E_j(\boldsymbol{r})dt \quad \text{(S13)}$$

where σ_{ij}($\mathbf{r}$) is the electrical conductivity tensor related to space; dt is the time of applied electric field.

The strain energy density is expressed by below:

$$f_{\text{strain}} = -\frac{\varphi_m^2}{2Y(\boldsymbol{r})} = -\frac{{\varepsilon_0}^2{\varepsilon_{\text{ij}}}^2(\boldsymbol{r}){E_i}^2(\boldsymbol{r})E_j(\boldsymbol{r})^2}{8Y(\boldsymbol{r})} \quad \text{(S14)}$$

where Y($\mathbf{r}$) is the Young' s modulus; φ_{m}=0.5$\varepsilon_0\varepsilon_{\text{r}}E^2$, is the mechanical compressive stress induced by an electric field.

The breakdown phase evolution can be described by a modified Allen-Cahn equation as follows:

$$\frac{\partial\eta(\boldsymbol{r},t)}{\partial t} = -L_0 H\begin{pmatrix}|f_{\text{elec}}| + |f_{\text{joule}}| \\ +|f_{\text{strain}}| - |f_{\text{critical}}|\end{pmatrix}\begin{bmatrix}\dfrac{\partial f_{\text{sep}}(\eta)}{\partial\eta(\boldsymbol{r},t)} + \dfrac{\partial f_{\text{grad}}(\boldsymbol{r})}{\partial\eta(\boldsymbol{r},t)} + \\ \dfrac{\partial f_{\text{elec}}(\boldsymbol{r})}{\partial\eta(\boldsymbol{r},t)} + \dfrac{\partial f_{\text{joule}}(\boldsymbol{r})}{\partial\eta(\boldsymbol{r},t)} + \dfrac{\partial f_{\text{strain}}(\boldsymbol{r})}{\partial\eta(\boldsymbol{r},t)}\end{bmatrix} \quad \text{(S15)}$$

where L_0 is the kinetic coefficient related to the interface mobility; $H(|f_{\text{elec}}|+|f_{\text{joule}}|+|f_{\text{strain}}|-|f_{\text{critical}}|)$ is the Heaviside unit step function. If $|f_{\text{elec}}| + |f_{\text{joule}}| + |f_{\text{strain}}| < |f_{\text{critical}}|$, $H(|f_{\text{elec}}| + |f_{\text{joule}}| + |f_{\text{strain}}| - |f_{\text{critical}}|)$= 0; If $|f_{\text{elec}}| + |f_{\text{joule}}| + |f_{\text{strain}}| > |f_{\text{critical}}|$, $H(|f_{\text{elec}}| + |f_{\text{joule}}| + |f_{\text{strain}}| - |f_{\text{critical}}|)$ = 1.

The driving force of phase separation is shown below:

$$\frac{\partial f_{\text{sep}}(\eta)}{\partial\eta(\boldsymbol{r},t)} = 2\alpha\eta(1-\eta)(1-2\eta) \quad \text{(S16)}$$

The relative dielectric constant related to space in PEI-BCF composites is described as follows:

$$\begin{aligned}\varepsilon_{\text{ij}}(\mathbf{r}) = {} & \eta^3(10-15\eta+6\eta^2)\varepsilon_{\text{ij}}^{\text{B}} + [1-\eta^3(10-15\eta+6\eta^2)]\times \\ & \left\{\rho^3(10-15\rho+6\rho^2)\varepsilon_{\text{ij}}^{\text{N}} + [1-\rho^3(10-15\rho+6\rho^2)]\varepsilon_{\text{ij}}^{\text{M}}\right\}\end{aligned} \quad \text{(S17)}$$

where ρ($\mathbf{r}$) is a non-evolving field variable, which is 1 in PEI-BCF phase and 0 in matrix; $\varepsilon_{\text{ij}}^{\text{B}}$ is the relative dielectric constant of the breakdown phase; $\varepsilon_{\text{ij}}^{\text{N}}$ is the relative dielectric constant of the PEI-BCF phase; $\varepsilon_{\text{ij}}^{\text{M}}$ is the relative dielectric constant of the matrix phase.

The driving force of electric term is shown below:

$$\frac{\partial f_{\text{elec}}}{\partial\eta(\mathbf{r},t)}=-15\eta^2(\eta-1)^2\varepsilon_0E_{\text{i}}(\mathbf{r})E_{\text{j}}(\mathbf{r})\times \left(\varepsilon_{\text{ij}}^{\text{B}}-\left\{\rho^3(10-15\rho+6\rho^2)\varepsilon_{\text{ij}}^{\text{N}}+[1-\rho^3(10-15\rho+6\rho^2)]\varepsilon_{\text{ij}}^{\text{M}}\right\}\right) \tag{S18}$$

The electrical conductivity related to space in PEI-BCF composites is described as follows:

$$\sigma_{\text{ij}}(\mathbf{r})=\eta^3(10-15\eta+6\eta^2)\sigma_{\text{ij}}^{\text{B}}+[1-\eta^3(10-15\eta+6\eta^2)]\times \left\{\rho^3(10-15\rho+6\rho^2)\sigma_{\text{ij}}^{\text{N}}+[1-\rho^3(10-15\rho+6\rho^2)]\sigma_{\text{ij}}^{\text{M}}\right\} \tag{S19}$$

where σ_{ij}^{B} is the electrical conductivity of the breakdown phase; σ_{ij}^{N} is the electrical conductivity of the PEI-BCF phase; σ_{ij}^{M} is the electrical conductivity of the matrix phase.

The driving force of Joule heat is shown below:

$$\frac{\partial f_{\text{joule}}}{\partial\eta(\mathbf{r},t)}=-30\eta^2(\eta-1)^2E_{\text{i}}(\mathbf{r})E_{\text{j}}(\mathbf{r})dt\times \left(\sigma_{\text{ij}}^{\text{B}}-\left\{\rho^3(10-15\rho+6\rho^2)\sigma_{\text{ij}}^{\text{N}}+[1-\rho^3(10-15\rho+6\rho^2)]\sigma_{\text{ij}}^{\text{M}}\right\}\right) \tag{S20}$$

The Young's modulus related to space in PEI-BCF phase is described as follows:

$$Y(\mathbf{r})=\eta^3(10-15\eta+6\eta^2)Y^{\text{B}}+[1-\eta^3(10-15\eta+6\eta^2)]\times \left\{\rho^3(10-15\rho+6\rho^2)Y^{\text{N}}+[1-\rho^3(10-15\rho+6\rho^2)]Y^{\text{M}}\right\} \tag{S21}$$

where Y^{B} is the Young's modulus of the breakdown phase; Y^{N} is the Young's modulus of the PEI-BCF phase; Y^{M} is the Young's modulus of the matrix phase.

The driving force of Joule heat is shown below:

$$\frac{\partial f_{\text{strain}}}{\partial\eta(\mathbf{r},t)}=-\frac{2\varepsilon_0^2\varepsilon_{\text{ij}}(\mathbf{r})E_{\text{i}}(\mathbf{r})^2E_{\text{j}}(\mathbf{r})^2}{8Y(\mathbf{r})}*\frac{\partial\varepsilon_{\text{ij}}(\mathbf{r})}{\partial\eta(\mathbf{r},t)}-\frac{1}{8Y(\mathbf{r})^2}\varepsilon_0^2\varepsilon_{\text{ij}}(\mathbf{r})^2E_{\text{i}}(\mathbf{r})^2E_{\text{j}}(\mathbf{r})^2*\frac{\partial Y(\mathbf{r})}{\partial\eta(\mathbf{r},t)} \tag{S22}$$

f_{critical} is a maximal energy density of composites which is a material constant related to the the position, as shown below:

$$f_{\text{critical}}=-\frac{1}{2}\varepsilon_0\varepsilon_rE_b{}^2 \tag{S23}$$

where E_b is the breakdown field strength of PEI-BCF composites.

The simulation parameters in phase-field model for electric-thermal-mechanical breakdown are shown in Table S5, Supporting Information.

Table S5. Parameters for electrical-thermal-mechanical breakdown model simulation.

Variables	Value	Unit	Content
ε_{ij}^{B}	10000	/	The relative dielectric constant of the breakdown phase
ε_{ij}^{N}	3.1	/	The relative dielectric constant of the PEI-BCF phase
ε_{ij}^{M}	3.1	/	The relative dielectric constant of the matrix phase
σ_{ij}^{B}	1×10^{-7}	$S\cdot m^{-1}$	The electrical conductivity of the breakdown phase
σ_{ij}^{N}	1×10^{-14}	$S\cdot m^{-1}$	The electrical conductivity of the PEI-BCF phase
σ_{ij}^{M}	1×10^{-12}	$S\cdot m^{-1}$	The electrical conductivity of the matrix phase
Y^{B}	1000	MPa	The Young's modulus of the breakdown phase
Y^{N}	2200	MPa	The Young's modulus of the PEI-BCF phase
Y^{M}	2000	MPa	The Young's modulus of the matrix phase
γ	1×10^{-9}	$J\cdot m^{-1}$	The gradient energy coefficient
E_b	678	$kV\cdot mm^{-1}$	The breakdown field strength of composites
E_b^{M}	546	$kV\cdot mm^{-1}$	The breakdown field strength of the matrix
α	1×10^{7}	$J\cdot m^{-3}$	The energy barrier of phase separation
L_0	1	$m^2\cdot s^{-1}\cdot N^{-1}$	The kinetic coefficient related to the interface mobility

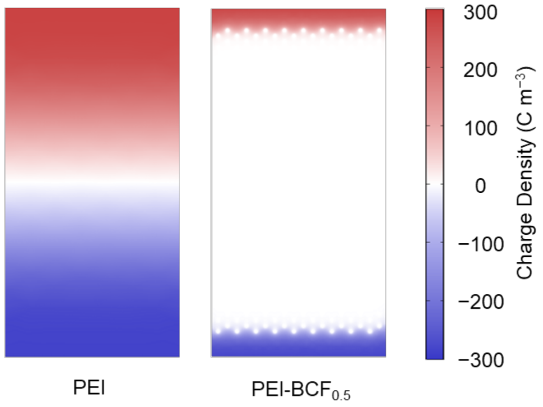

Figure S22. Simulated space charge density distribution of PEI and PEI-$BCF_{0.5}$ at 200 °C and 500 MV m^{-1}.

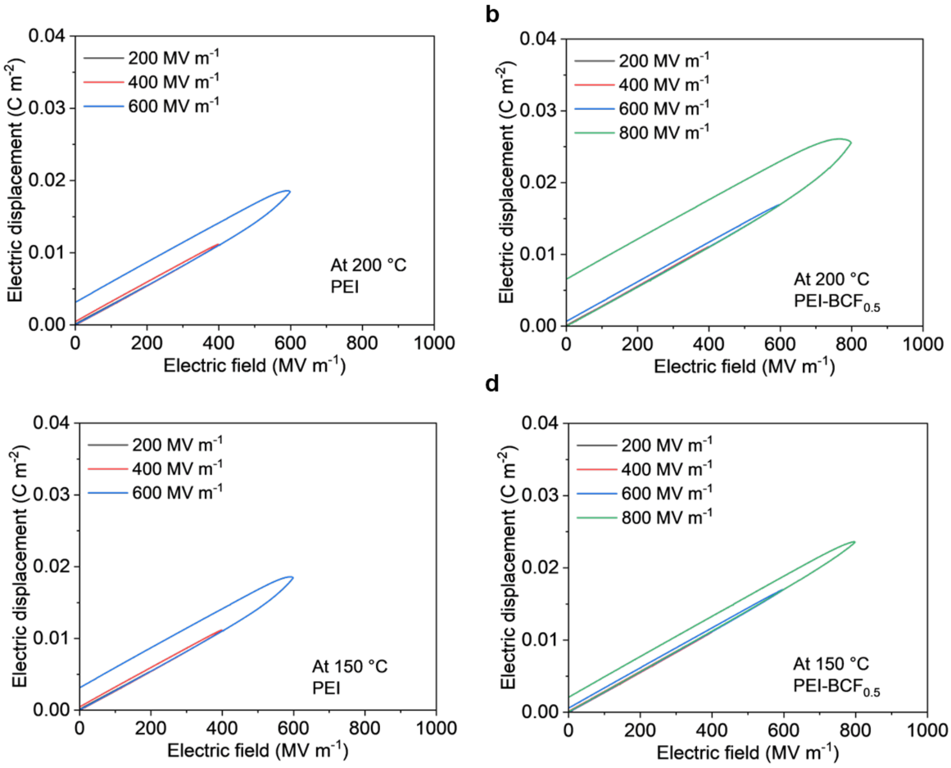

Figure S23. D–E curves of PEI and PEI-BCF$_{0.5}$ at (a), (b) 200 °C and (c), (d)150 °C.

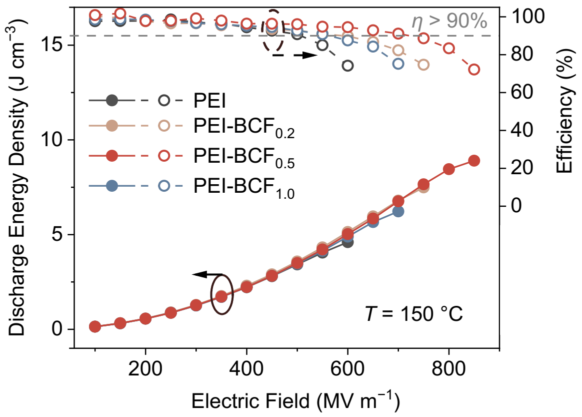

Figure S24. Discharged energy density and charge-discharge efficiency of pure PEI and PEI-BCF$_n$ at 150 °C.

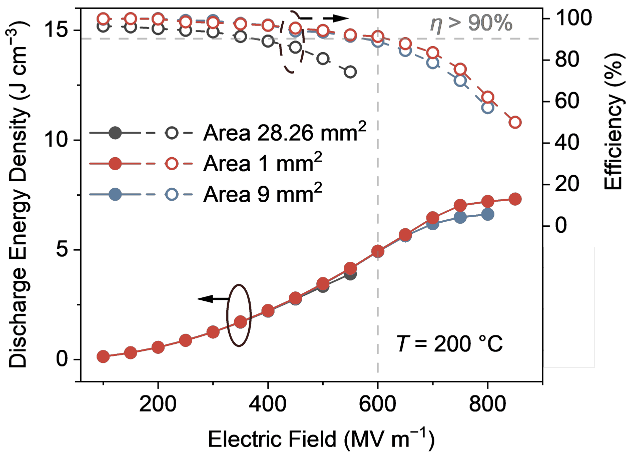

Figure S25. Discharged energy density and charge-discharge efficiency of PEI-BCF0.5 films with varying electrode areas at 200 °C.

Table S6. Comparison of energy storage performance.

Materials	Energy storage performance	Refs
PEI/5 vol% SiO_2	150 °C: $U_{d\text{-}MAX}$ ~6.3 J cm^{-3}, $U_{d\text{-}90}$ ~6.3 J cm^{-3}, E_b ~608 MV m^{-1}	[S1]
PEI/0.75 wt% PMHT	150 °C: $U_{d\text{-}MAX}$ ~5.1 J cm^{-3}, $U_{d\text{-}90}$ ~4.1 J cm^{-3}<br>200 °C: $U_{d\text{-}MAX}$ ~3.3 J cm^{-3}, $U_{d\text{-}90}$ ~2.0 J cm^{-3}, E_b ~458 MV m^{-1}	[S2]
PEI/h-BN	150 °C: $U_{d\text{-}90}$ ~2.2 J cm^{-3}<br>200 °C: $U_{d\text{-}90}$ ~1.2 J cm^{-3}	[S3]
PEI/HEnf	150 °C: $U_{d\text{-}MAX}$ ~6.46 J cm^{-3}, $U_{d\text{-}90}$ ~2.5 J cm^{-3}, E_b ~590 MV m^{-1}	[S4]
PEI/0.5 wt% HAP	150 °C: $U_{d\text{-}MAX}$ ~5.2 J cm^{-3}, $U_{d\text{-}90}$ ~5.14 J cm^{-3}, E_b ~645 MV m^{-1}<br>200 °C: $U_{d\text{-}MAX}$ ~4.8 J cm^{-3}, $U_{d\text{-}90}$ ~3.1 J cm^{-3}, E_b ~530 MV m^{-1}	[S5]
PEI/0.5 NTCDA	150 °C: $U_{d\text{-}MAX}$ ~5.14 J cm^{-3}, $U_{d\text{-}90}$ ~5.14 J cm^{-3}, E_b ~630 MV m^{-1}<br>200 °C: $U_{d\text{-}MAX}$ ~3.8 J cm^{-3}, $U_{d\text{-}90}$ ~3.2 J cm^{-3}, E_b ~510 MV m^{-1}	[S6]
PEI/ZrO_2@Al_2O_3	150 °C: $U_{d\text{-}MAX}$ ~5.2 J cm^{-3}, $U_{d\text{-}90}$ ~3.8 J cm^{-3}, E_b ~585 MV m^{-1}	[S7]
PEI-O-AOC	200 °C: $U_{d\text{-}MAX}$ ~6.8 J cm^{-3}, $U_{d\text{-}90}$ ~6.8 J cm^{-3}, E_b ~556 MV m^{-1}	[S8]
P-PEI	150 °C: $U_{d\text{-}MAX}$ ~5 J cm^{-3}, $U_{d\text{-}90}$ ~4.62 J cm^{-3}, E_b ~645 MV m^{-1}<br>200 °C: $U_{d\text{-}MAX}$ ~3.5 J cm^{-3}, $U_{d\text{-}90}$ ~3.2 J cm^{-3}, E_b ~563 MV m^{-1}	[S9]
PEI/0.5 wt% UIO-66	150 °C: $U_{d\text{-}MAX}$ ~5.9 J cm^{-3}, $U_{d\text{-}90}$ ~5.9 J cm^{-3}, E_b ~617 MV m^{-1}<br>200 °C: $U_{d\text{-}MAX}$ ~3.7 J cm^{-3}, $U_{d\text{-}90}$ ~2.4 J cm^{-3}, E_b ~516 MV m^{-1}	[S10]
PEI/ITIC-Cl-0.2%	150 °C: $U_{d\text{-}MAX}$ ~4.8 J cm^{-3}, $U_{d\text{-}90}$ ~4.30 J cm^{-3}<br>200 °C: $U_{d\text{-}MAX}$ ~3.90 J cm^{-3}, $U_{d\text{-}90}$ ~3.10 J cm^{-3}, E_b ~520 MV m^{-}	[S11]
PEI/20% PESU	150 °C: $U_{d\text{-}MAX}$ ~5.4 J cm^{-3}, $U_{d\text{-}90}$ ~2.36 J cm^{-3}, E_b ~570 MV m^{-1}<br>200 °C: $U_{d\text{-}MAX}$ ~2.90 J cm^{-3}, $U_{d\text{-}90}$ ~0.94 J cm^{-3}, E_b ~480 MV m^{-1}	[S12]
PEI/0.5 vol% PCBM	150 °C: $U_{d\text{-}MAX}$ ~4.8 J cm^{-3}, $U_{d\text{-}90}$ ~4.6 J cm^{-3}, E_b ~664 MV m^{-1}<br>200 °C: $U_{d\text{-}MAX}$ ~3.2 J cm^{-3}, $U_{d\text{-}90}$ ~3.0 J cm^{-3}, E_b ~649 MV m^{-1}	[S13]
PEI/0.75 vol% DPDI	150 °C: $U_{d\text{-}MAX}$ ~4.4 J cm^{-3}, $U_{d\text{-}90}$ ~4.0 J cm^{-3}, E_b ~646 MV m^{-1}<br>200 °C: $U_{d\text{-}MAX}$ ~3.0 J cm^{-3}, $U_{d\text{-}90}$ ~2.5 J cm^{-3}, E_b ~625 MV m^{-1}	[S13]
PEI/0.25 vol% ITIC	150 °C: $U_{d\text{-}MAX}$ ~4.2 J cm^{-3}, $U_{d\text{-}90}$ ~3.4 J cm^{-3}, E_b ~658 MV m^{-1}<br>200 °C: $U_{d\text{-}MAX}$ ~2.7 J cm^{-3}, $U_{d\text{-}90}$ ~2.2 J cm^{-3}, E_b ~638 MV m^{-1}	[S13]
PEI/0.2 wt% PWNS	150 °C: $U_{d\text{-}MAX}$ ~7.8 J cm^{-3}, $U_{d\text{-}90}$ ~7.2 J cm^{-3}<br>200 °C: $U_{d\text{-}MAX}$ ~6.7 J cm^{-3}, $U_{d\text{-}90}$ ~4.1 J cm^{-3}, E_b ~650 MV m^{-1}	[S14]
PEI/5 wt% TE	150 °C: $U_{d\text{-}MAX}$ ~6.2 J cm^{-3}, $U_{d\text{-}90}$ ~5.5 J cm^{-3}, E_b ~651 MV m^{-1}<br>200 °C: $U_{d\text{-}MAX}$ ~5.8 J cm^{-3}, $U_{d\text{-}90}$ ~4.0 J cm^{-3}, E_b ~638 MV m^{-1}	[S15]
PEI/5 wt% PER	150 °C: $U_{d\text{-}MAX}$ ~6.0 J cm^{-3}, $U_{d\text{-}90}$ ~5.4 J cm^{-3}, E_b ~639 MV m^{-1}<br>200 °C: $U_{d\text{-}MAX}$ ~5.4 J cm^{-3}, $U_{d\text{-}90}$ ~3.8 J cm^{-3}, E_b ~621 MV m^{-1}	[S15]
PEI/8 wt% TPE	150 °C: $U_{d\text{-}MAX}$ ~5.6 J cm^{-3}, $U_{d\text{-}90}$ ~5.1 J cm^{-3}, E_b ~622 MV m^{-1}<br>200 °C: $U_{d\text{-}MAX}$ ~4.1 J cm^{-3}, $U_{d\text{-}90}$ ~3.0 J cm^{-3}, E_b ~590 MV m^{-1}	[S15]
PEI/UV-Al_2O_3	150 °C: $U_{d\text{-}MAX}$ ~5.91 J cm^{-3}, $U_{d\text{-}90}$ ~5.69 J cm^{-3}, E_b ~622 MV m^{-1}	[S16]

	200 °C: $U_{d\text{-}MAX}$ ~4.2 J cm^{-3}, $U_{d\text{-}90}$ ~3.59 J cm^{-3}, E_b ~566 MV m^{-1}	
PEI/H-Al_2O_3	150 °C: $U_{d\text{-}MAX}$ ~6.57 J cm^{-3}, $U_{d\text{-}90}$ ~6.57 J cm^{-3}, E_b ~608 MV m^{-1}<br>200 °C: $U_{d\text{-}MAX}$ ~4.77 J cm^{-3}, $U_{d\text{-}90}$ ~3.8 J cm^{-3}, E_b ~528 MV m^{-1}	[S17]
PEI/ 0.5 wt% C_{18}	200 °C: $U_{d\text{-}MAX}$ ~5.2 J cm^{-3}, $U_{d\text{-}90}$ ~5.2 J cm^{-3}	[S18]
PEI/ 0.5 wt% Crown(III)	200 °C: $U_{d\text{-}MAX}$ ~5.3 J cm^{-3}, $U_{d\text{-}90}$ ~5.3 J cm^{-3}	[S18]
PEI/BMI PND	150 °C: $U_{d\text{-}MAX}$ ~8.1 J cm^{-3}, $U_{d\text{-}90}$ ~7.8 J cm^{-3}, E_b ~709 MV m^{-1}<br>200 °C: $U_{d\text{-}MAX}$ ~6.1 J cm^{-3}, $U_{d\text{-}90}$ ~5.8 J cm^{-3}, E_b ~612 MV m^{-1}	[S19]
PEI-g-TOC	150 °C: $U_{d\text{-}MAX}$ ~8.1 J cm^{-3}, $U_{d\text{-}90}$ ~7.53 J cm^{-3}, E_b ~657 MV m^{-1}<br>200 °C: $U_{d\text{-}MAX}$ ~5 J cm^{-3}, $U_{d\text{-}90}$ ~4.55 J cm^{-3}, E_b ~553 MV m^{-1}	[S20]
PEI/8 wt% DG	150 °C: $U_{d\text{-}MAX}$ ~5.48 J cm^{-3}, $U_{d\text{-}90}$ ~5.48 J cm^{-3}, E_b ~600 MV m^{-1}<br>200 °C: $U_{d\text{-}MAX}$ ~4.1 J cm^{-3}, $U_{d\text{-}90}$ ~4.1 J cm^{-3}, E_b ~550 MV m^{-1}	[S21]
PEI/0.3 wt% PAPD-UV	150 °C: $U_{d\text{-}MAX}$ ~5.2 J cm^{-3}, $U_{d\text{-}90}$ ~5.2 J cm^{-3}, E_b ~630 MV m^{-1}<br>200 °C: $U_{d\text{-}MAX}$ ~3.5 J cm^{-3}, $U_{d\text{-}90}$ ~2.6 J cm^{-3}, E_b ~510 MV m^{-1}	[S22]
PEI/E-c-SiO_2	150 °C: $U_{d\text{-}MAX}$ ~6.81 J cm^{-3}, $U_{d\text{-}90}$ ~6.81 J cm^{-3}, E_b ~649 MV m^{-1}<br>200 °C: $U_{d\text{-}MAX}$ ~4.98 J cm^{-3}, $U_{d\text{-}90}$ ~4.26 J cm^{-3}, E_b ~601 MV m^{-1}	[S23]
PEI/0.4 vol% F4TCNQ	150 °C: $U_{d\text{-}MAX}$ ~5.00 J cm^{-3}, $U_{d\text{-}90}$ ~4.86 J cm^{-3}, E_b ~632 MV m^{-1}	[S24]
PEI/0.8 vol% 4NB	150 °C: $U_{d\text{-}MAX}$ ~5.21 J cm^{-3}, $U_{d\text{-}90}$ ~5.21 J cm^{-3}, E_b ~646 MV m^{-1}	[S24]
PEI/AOC-0.5	150 °C: $U_{d\text{-}MAX}$ ~7.7 J cm^{-3}, $U_{d\text{-}90}$ ~7.1 J cm^{-3}, E_b ~630 MV m^{-1}<br>200 °C: $U_{d\text{-}MAX}$ ~6 J cm^{-3}, $U_{d\text{-}90}$ ~4.4 J cm^{-3}, E_b ~582 MV m^{-1}	[S25]
PEI-$BCF_{0.5}$	150 °C: $U_{d\text{-}MAX}$ ~8.9 J cm^{-3}, $U_{d\text{-}90}$ ~6.8 J cm^{-3}, E_b ~701 MV m^{-1}<br>200 °C: $U_{d\text{-}MAX}$ ~7.3 J cm^{-3}, $U_{d\text{-}90}$ ~5.3 J cm^{-3}, E_b ~678 MV m^{-1}	This work

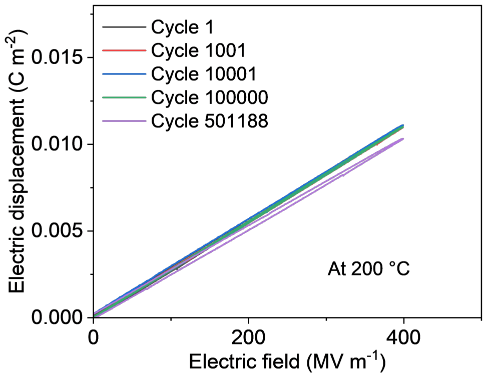

Figure S26. *D*–*E* curves under various aging cycles of PEI-$BCF_{0.5}$ at 200 °C.

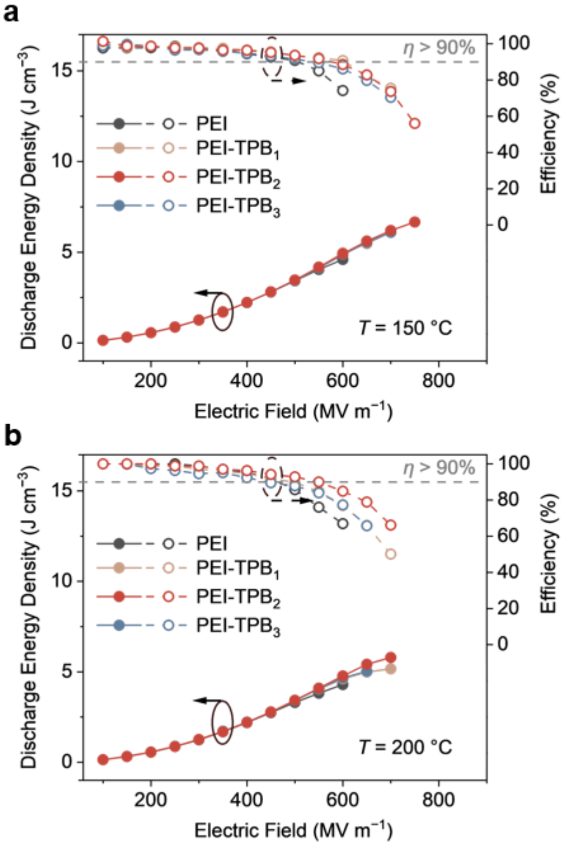

Figure S27. Discharged energy density and charge-discharge efficiency of PEI-TPB_n (where n = 1, 2 and 3 indicates TPB weight percentage, wt%) at (a) 150 °C and (b) 200 °C.

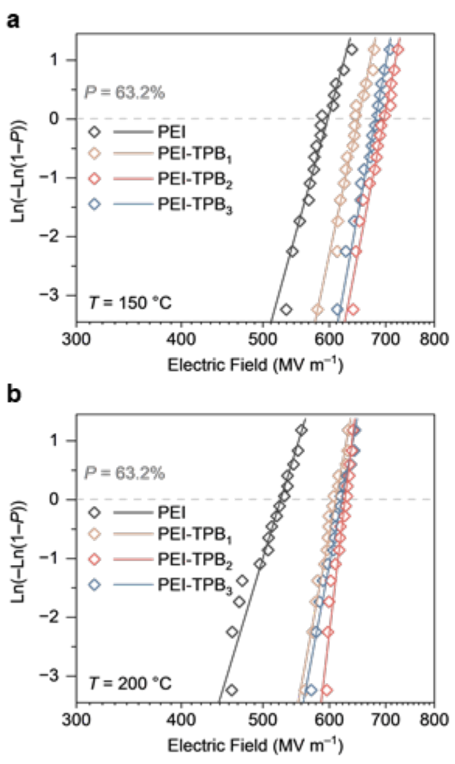

Figure S28. Weibull breakdown plots of PEI-TPB$_n$ at (a) 150 °C and (b) 200 °C.

Table S7. Weibull breakdown parameters of PEI and PEI-TPB$_n$ films at 150 and 200 °C.

Materials	150 °C		200 °C	
	E_b (MV m^{-1})	$β$	E_b (MV m^{-1})	$β$
PEI	597	22	525	20
PEI-TPB$_1$	649	29	610	33
PEI-TPB$_2$	696	32	626	52
PEI-TPB$_3$	680	33	620	32

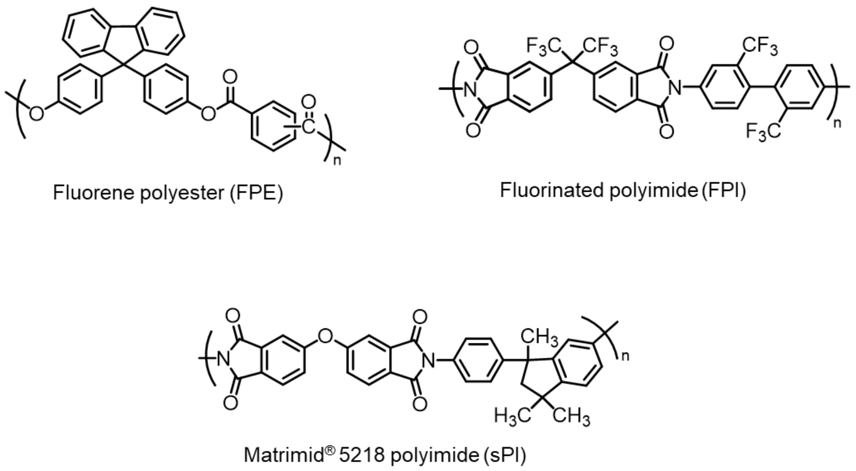

Figure S29. Chemical structures of other commercial carbonyl-containing polymers tested in this work.

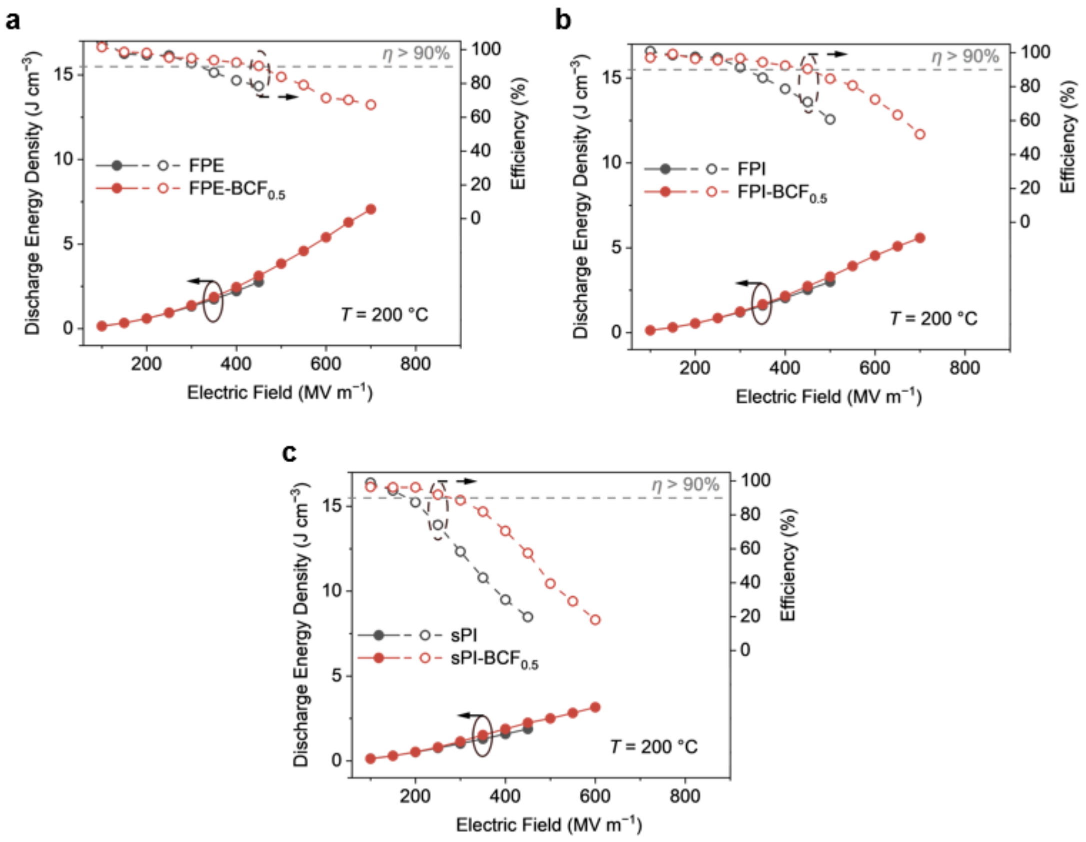

Figure S30. Discharged energy density and charge-discharge efficiency of (a) FPE-$BCF_{0.5}$, (b) FPI-$BCF_{0.5}$ and (c) sPI-$BCF_{0.5}$ at 200 °C.

References

[S1] B. Sun, P. Hu, X. Ji, et al., "Excellent Stability in Polyetherimide/SiO2 Nanocomposites with Ultrahigh Energy Density and Discharge Efficiency at High Temperature," *Small* 18, no. 28 (2022): 2202421. https://doi.org/10.1002/smll.202202421.
[S2] C. F. Yan, Y. T. Wan, H. P. Long, et al., "Improved Capacitive Energy Storage at High Temperature via Constructing Physical Cross-Link and Electron-Hole Pairs Based on P-Type Semiconductive Polymer Filler," *Advanced Functional Materials* 34, no. 8 (2024): 2312238. https://doi.org/10.1002/adfm.202312238.
[S3] A. Azizi, M. R. Gadinski, Q. Li, et al., "High-Performance Polymers Sandwiched with Chemical Vapor Deposited Hexagonal Boron Nitrides as Scalable High-Temperature Dielectric Materials," *Advanced Materials* 29, no. 35 (2017): 1701864. https://doi.org/10.1002/adma.201701864.
[S4] L. Dou, B. Yang, S. Lan, et al., "High‐Entropy‐Nanofibers Enhanced Polymer Nanocomposites for High‐Performance Energy Storage," *Advanced Energy Materials* 13, no. 11 (2023. https://doi.org/10.1002/aenm.202203925.
[S5] M. Z. Yang, F. Yuan, W. X. Shi, et al., "Sub-Nanowires Boost Superior Capacitive Energy Storage Performance of Polymer Composites at High Temperatures," *Advanced Functional Materials* 33, no. 12 (2023): 2214100. https://doi.org/10.1002/adfm.202214100.
[S6] B. Zhang, X. M. Chen, Z. Pan, et al., "Superior High-Temperature Energy Density in Molecular Semiconductor/Polymer All-Organic Composites," *Advanced Functional Materials* 33, no. 5 (2023): 2210050. https://doi.org/10.1002/adfm.202210050.
[S7] L. L. Ren, H. Li, Z. L. Xie, et al., "High-Temperature High-Energy-Density Dielectric Polymer Nanocomposites Utilizing Inorganic Core-Shell Nanostructured Nanofillers," *Advanced Energy Materials* 11, no. 28 (2021): 2101297. https://doi.org/10.1002/aenm.202101297.
[S8] M. Yang, S. Wang, J. Fu, et al., "Quantum Size Effect to Induce Colossal High-Temperature Energy Storage Density and Efficiency in Polymer/Inorganic Cluster Composites," *Advanced Materials* 35, no. 30 (2023): 2301936. https://doi.org/10.1002/adma.202301936.
[S9] J. Y. Pei, J. Zhu, L. J. Yin, et al., "Flexible High-Temperature Polymer Dielectrics Induced by Ultraviolet Radiation for High Efficient Energy Storage," *Advanced Functional Materials* 34, no. 41 (2024): 2316869. https://doi.org/10.1002/adfm.202316869.
[S10] N. Zhang, H. Zhao, C. Zhang, et al., "Ultrafine MOF as charge trap enables superior high-temperature energy storage performance in polyetherimide composites dielectrics," *Chemical Engineering Journal* 508 (2025). https://doi.org/10.1016/j.cej.2025.161063.
[S11] Y. Zhou, Y. J. Zhu, W. H. Xu, et al., "Molecular Trap Engineering Enables Superior High-Temperature Capacitive Energy Storage Performance in All-Organic Composite at 200 °C," *Advanced Energy Materials* 13, no. 11 (2023): 2203961. https://doi.org/10.1002/aenm.202203961.
[S12] C. H. Zhang, X. Tong, T. D. Zhang, et al., "Constructing a dual gradient structure of energy level gradient and concentration gradient to significantly improve the high-temperature energy storage performance of all organic composite dielectrics," *Chemical Engineering Journal* 491, (2024): 151634. https://doi.org/10.1016/j.cej.2024.151634.
[S13] C. Yuan, Y. Zhou, Y. Zhu, et al., "Polymer/molecular semiconductor all-organic composites for high-temperature dielectric energy storage," *Nature Communications* 11, no. 1 (2020): 3919. https://doi.org/10.1038/s41467-020-17760-x.
[S14] M. Z. Yang, H. Y. Li, J. Wang, et al., "Roll-to-roll fabricated polymer composites filled with subnanosheets exhibiting high energy density and cyclic stability at 200 °C," *Nature Energy* 9, no. 2 (2024): 143. https://doi.org/10.1038/s41560-023-01416-3.
[S15] M. Yang, L. Zhou, X. Li, et al., "Polyimides Physically Crosslinked by Aromatic Molecules Exhibit Ultrahigh Energy Density at 200 °C," *Advanced Materials* 35, no. 35 (2023): 2302392. https://doi.org/10.1002/adma.202302392.
[S16] M. Yang, Z. Wang, Y. Zhao, et al., "Unifying and Suppressing Conduction Losses of Polymer Dielectrics for Superior High-Temperature Capacitive Energy Storage," *Adv Mater* 36, no. 52 (2024): 2309640. https://doi.org/10.1002/adma.202309640.
[S17] M. H. Yang, Y. L. Zhao, Z. P. Wang, et al., "Surface ion-activated polymer composite dielectrics for superior high-temperature capacitive energy storage," *Energy & Environmental Science* 17, no. 4 (2024): 1592. https://doi.org/10.1039/d3ee03644h.
[S18] R. Wang, Y. Zhu, S. Huang, et al., "Dielectric polymers with mechanical bonds for high-temperature capacitive energy storage," *Nature Materials* 24, no. 7 (2025): 1074. https://doi.org/10.1038/s41563-025-02130-z.
[S19] Q. Zhang, Q. Xie, T. Wang, et al., "Scalable all polymer dielectrics with self-assembled nanoscale multiboundary exhibiting superior high temperature capacitive performance," *Nature Communications* 15, no. 1 (2024): 9351. https://doi.org/10.1038/s41467-024-53674-8.

[S20] S. Zhao, W. Peng, L. Zhou, et al., "Metal-organic cage crosslinked nanocomposites with enhanced high-temperature capacitive energy storage performance," *Nature Communications* 16, no. 1 (2025): 769. https://doi.org/10.1038/s41467-025-56069-5.
[S21] M. Yang, W. Ren, Z. Jin, et al., "Enhanced high-temperature energy storage performances in polymer dielectrics by synergistically optimizing band-gap and polarization of dipolar glass," *Nature Communications* 15, no. 1 (2024): 8647. https://doi.org/10.1038/s41467-024-52791-8.
[S22] Q. T. Wang, J. L. Ding, W. Jiang, et al., "Roll-to-Roll Production of High-Performance All-Organic Polymer Nanocomposites for High-Temperature Capacitive Energy Storage," *Advanced Functional Materials* 35, no. 6 (2025): 2414616. https://doi.org/10.1002/adfm.202414616.
[S23] Z. P. Wang, Y. L. Zhao, M. H. Yang, et al., "Surface Strengthening of Polymer Composite Dielectrics for Superior High-Temperature Capacitive Energy Storage," *Advanced Energy Materials* 15, no. 19 (2025): 2405411. https://doi.org/10.1002/aenm.202405411.
[S24] Z. T. Meng, T. D. Zhang, C. H. Zhang, et al., "Optimizing Energy Storage Performance in Polymer Dielectrics through Dual Strategies: Constructing "Peaked" Barrieras and Enhancing Carrier Scattering," *Advanced Functional Materials* 34, no. 39 (2024): 2403402. https://doi.org/10.1002/adfm.202403402.
[S25] Z. Pan, Y. Cheng, Z. Li, et al., "Aluminum macrocycles induced superior high-temperature capacitive energy storage for polymer-based dielectrics via constructing charge trap rings," *Energy & Environmental Science*, (2025). https://doi.org/10.1039/d4ee05689b.
